# Paradoxical activation of the protein kinase-transcription factor ERK5 by ERK5 kinase inhibitors

**DOI:** 10.1038/s41467-020-15031-3

**Published:** 2020-03-13

**Authors:** Pamela A. Lochhead, Julie A. Tucker, Natalie J. Tatum, Jinhua Wang, David Oxley, Andrew M. Kidger, Victoria P. Johnson, Megan A. Cassidy, Nathanael S. Gray, Martin E. M. Noble, Simon J. Cook

**Affiliations:** 1 Signalling Laboratory, The Babraham Institute, Babraham Research Campus, Cambridge, CB22 3AT UK; 2 York Biomedical Research Institute and Department of Biology, University of York, York, YO10 5DD UK; 3 CRUK Newcastle Drug Discovery Unit, Newcastle University Centre for Cancer, Newcastle University, Newcastle, NE2 4HH UK; 4 Department of Cancer Biology, Dana-Farber Cancer Institute, Boston, MA 02215 USA; 5 Department of Biological Chemistry and Molecular Pharmacology, Harvard Medical School, Boston, MA 02115 USA; 6 Present Address: Institute of Cancer Research, Chester Beatty Laboratories, 237 Fulham Road, London, SW3 6JB UK

**Keywords:** Biochemistry, Enzyme mechanisms, Cancer, Cell signalling, Cellular imaging

## Abstract

The dual protein kinase-transcription factor, ERK5, is an emerging drug target in cancer and inflammation, and small-molecule ERK5 kinase inhibitors have been developed. However, selective ERK5 kinase inhibitors fail to recapitulate ERK5 genetic ablation phenotypes, suggesting kinase-independent functions for ERK5. Here we show that ERK5 kinase inhibitors cause paradoxical activation of ERK5 transcriptional activity mediated through its unique C-terminal transcriptional activation domain (TAD). Using the ERK5 kinase inhibitor, Compound **26** (ERK5-IN-1), as a paradigm, we have developed kinase-active, drug-resistant mutants of ERK5. With these mutants, we show that induction of ERK5 transcriptional activity requires direct binding of the inhibitor to the kinase domain. This in turn promotes conformational changes in the kinase domain that result in nuclear translocation of ERK5 and stimulation of gene transcription. This shows that both the ERK5 kinase and TAD must be considered when assessing the role of ERK5 and the effectiveness of anti-ERK5 therapeutics.

## Abstract (web summary)

Selective ERK5 inhibitors target ERK5 kinase activity, but they do not phenocopy the effects of ERK5 genetic depletion. Here, the authors demonstrate that the direct interaction of these inhibitors to ERK5 kinase domain induces conformational changes that promote ERK5 nuclear translocation and transcriptional activities.

## Introduction

**Fig. 1:**
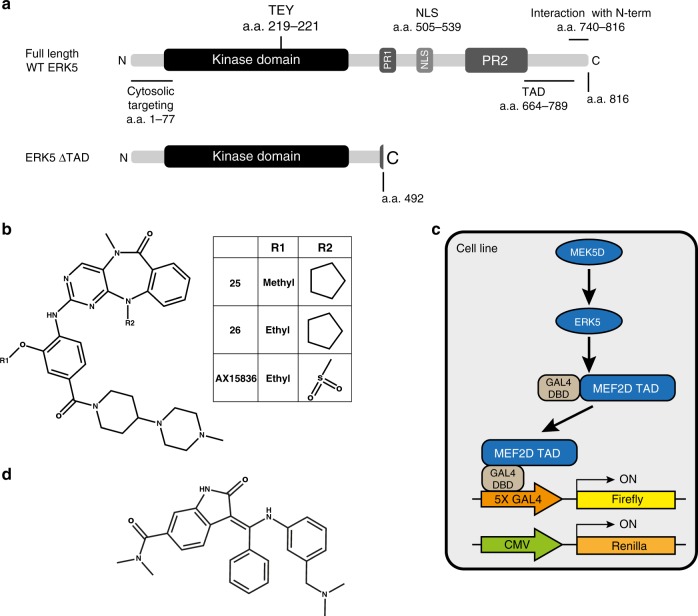
Schematic diagrams of the reagents used in this study. **a** Schematic diagram of ERK5 (full length) and ERK5 ΔTAD, which lacks the C-terminal extension. Functional domains with amino acid positions are: cytosolic targeting domain (1–77), kinase domain (48–383), activation-loop TEY motif (219–221), proline rich domain (PR) 1 (434–485) and 2 (578–701), nuclear localisation signal (NLS) (505–539), minimal transactivation domain (TAD) (664–789), and the N-terminal interaction domain (740-816). **b** Chemical structures of ERK5i: compounds **25**, **26** and AX15836. **c** Schematic representation of the ERK5:MEF2D luciferase assay. **d** Chemical structure of the MEK5i, BIX02189. Structures were drawn using ChemDraw v16.0.

Extracellular signal-regulated kinase 5 (ERK5, also known as Big MAP Kinase or BMK1)^1,2^ is a member of the mitogen-activated protein kinase (MAPK) family, which also includes ERK1/2^3^, the JNKs^4^ and the p38 kinases^5^. Like ERK1/2, ERK5 is the effector kinase of a three-tiered MAPK pathway, comprising MEKK2 and MEKK3 (the MKKKs), MEK5 (MKK) and finally ERK5 (MAPK). ERK5 is encoded by the *MAPK7* gene and includes an N-terminal kinase domain that shares 50% identity with ERK2^1,2^. However, it also contains a large, unique C-terminal extension that includes a nuclear localisation signal (NLS) and a transcriptional activation domain (TAD) (Fig. 1a)^6^. The ERK5 pathway is activated by mitogens^7^, agonists of the Toll-like receptor-2^8^ and cellular stresses^9^. Upon cellular stimulation, activated MEK5 phosphorylates the TEY motif in the ERK5 activation-loop, leading to activation of its kinase domain^10^. The ERK5 C-terminus also becomes auto-phosphorylated and promotes ERK5 translocation from the cytosol to the nucleus^11,12^, where ERK5 has been shown to interact with MEF2 transcription factors such as MEF2D^7,13,14^. The C-terminus can also be regulated by other protein kinases, including ERK1/2^15^ and CDK1^16,17^, which phosphorylate C-terminal residues independently of ERK5 kinase activity. Thus, the C-terminus mediates some of the effects of ERK5 kinase activity and integrates signals from other pathways.

There is a growing appreciation of the role that ERK5 signalling plays in some diseases, most notably in inflammation and cancer. For example, ERK5 plays a pro-inflammatory role in human endothelial cells and monocytes^8,18^ and ERK5 inhibition exerts an anti-inflammatory effect. ERK5 is also implicated as a mediator of inflammation-driven cancer^19,20^. Finally, a range of studies have suggested that ERK5 signalling promotes cell proliferation, cell survival and motility and invasion^21–23^. While MEK5 or ERK5 mutations are rare in cancer, these components are sometimes over-expressed; indeed, *MAPK7* is amplified in hepatocellular carcinoma (HCC)^24^, although it appears not to drive HCC cell proliferation^25^. In addition, ERK5 is activated in melanoma cells with BRAF mutations^26^ and such cells can acquire resistance to the BRAF inhibitor vemurafenib by increasing ERK5 phosphorylation^27^. Thus, ERK5 may drive key cancer hallmarks and promote resistance to other targeted agents.

These observations have prompted commercial and academic MEK5 or ERK5 drug discovery programmes in the hope of developing novel anti-inflammatory or anti-cancer therapeutics. Commercial MEK5 or ERK5 inhibitor programmes include ActivX, Kyorin Pharmaceutical Co.^28^, Bayer AG^29^, Boehringer Ingelheim^30^ and AstraZeneca^31^. The first ERK5 inhibitor (ERK5i) to be described was XMD8-92 from the Dana-Farber Cancer Institute and the Scripps Research Institute, USA^32,33^. Using the KiNativ method^34,35^ XMD8-92 was found to inhibit ERK5 with an IC_50_ of 1.5 µM, being tenfold more selective than its most potent off-target kinases. In cells both siRNA knockdown of ERK5 or XMD8-92 treatment caused an increase in p21^CIP1^ gene-expression, and XMD8-92 or expression of a dominant-negative ERK5 (AEF: where the activation-loop TEY phosphorylation sites are mutated to AEF) decreased tumour growth^32,33^. The subsequent ERK5 inhibitors, compounds **25** and **26** (Fig. 1b), were generated through a collaboration between the Dana-Farber Cancer Institute, USA, and the Structural Genomics Consortium in Oxford, UK and others. Compounds **25** and **26** (also known as ERK5-IN-1 and XMD17-109) are more potent than XMD8-92, and compound **26** represents a further improvement in selectivity over XMD8-92^36,37^. Recently, AX15836 (Fig. 1b), a new, more potent and more selective ERK5 inhibitor was developed at ActivX Biosciences Inc, USA and Kyorin Pharmaceutical Co., Japan^28^. Notably, AX15836 did not share with XMD8-92 the ability to block immune responses or inhibit tumour cell proliferation. This led to the discovery that XMD8-92 derived most of its biological activity from off-target interactions with bromodomain-containing proteins such as BRD4. Thus, dual ERK5-BRD inhibitors, such as XMD8-92 and compound **26**, are no longer suitable for dissecting the contribution that ERK5 plays within the cell. Selective ERK5 inhibition, without activity against BRD proteins, can be achieved with AX15836 or the new ERK5i compound **46**^38^ making them useful small molecules for interrogating the cellular roles of ERK5. Critically, AX15836 failed to replicate the effects of genetic ablation of ERK5 on cytokine release from immune cells or cell proliferation^28^. The authors suggested that this disconnect between ERK5 kinase inhibition and ablation of ERK5 expression was evidence of kinase-independent effects of ERK5 signalling^28^.

Here, we provide an important new insight into this by showing that a variety of ERK5 inhibitors, including compounds **25** and **26** but also AX15836, while being very effective at inhibiting the ERK5 kinase domain cause a paradoxical activation of the ERK5 transcriptional activation domain and drive ERK5-dependent gene transcription. Critically our study suggests that both the ERK5 kinase domain and the ERK5 TAD must be considered when validating the role of ERK5 in biological processes, in diseases where ERK5 is implicated and in designing and assessing the effectiveness of anti-ERK5 therapeutics.

## Results

### ERK5i induce transcriptional activity in the ERK5:MEF2D reporter system independently of kinase activity

The paucity of well-validated ERK5 substrates led us to use an ERK5-dependent transient transfection reporter assay to determine the ‘in cell’ IC_50_ of small-molecule ERK5 kinase inhibitors (ERK5i). This system (Fig. 1c) comprised: (i) a constitutively active form of the ERK5 kinase MEK5, MEK5D (where the regulatory activation-loop phosphorylation sites in MEK5 are mutated to phospho-mimetic aspartic acid, producing a constitutively active form of MEK5), to phosphorylate and activate ERK5; (ii) HA-tagged, full length human ERK5; (iii) a fusion protein of the transactivation domain (TAD) of MEF2D and the DNA binding domain of the yeast transcription factor GAL4 (MEF2D is one of the few validated ERK5 substrates and interacting proteins^6,7,14,39^); (iv) a luciferase reporter construct with upstream multimerised GAL4 binding sites and (v) a Renilla reporter construct that is regulated by the constitutive CMV promoter as a transfection control. In this system, MEK5D phosphorylates the activation-loop of ERK5 causing activation of its kinase domain. The kinase domain of ERK5 then phosphorylates the MEF2D TAD leading to activation, thereby driving luciferase expression, which serves as a readout of ERK5 kinase activity. We previously validated this system and used it to monitor ERK5 activity^25,40,41^. The ERK5 inhibitors compound **26**, compound **25**^32,37^, AX15836^28^ (Fig. 1b) and MEK5 inhibitor BIX02189^30^ (Fig. 1d) were used in our studies.

**Fig. 2:**
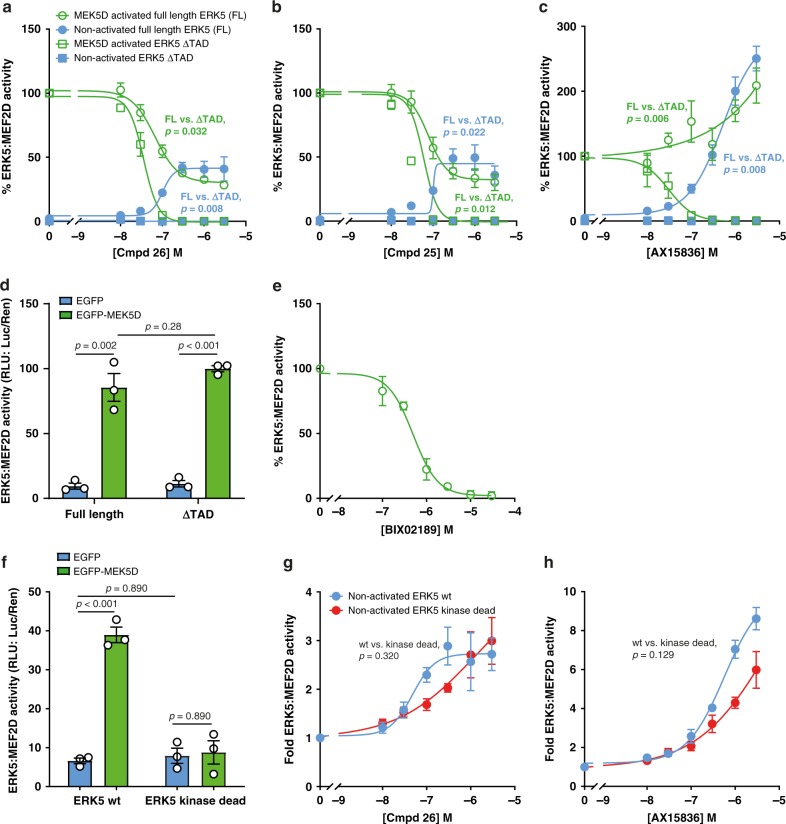
ERK5i induce transcriptional activity in the ERK5:MEF2D reporter system independently of kinase activity. **a**, **b**, **c** and **e** HEK293 cells were transfected with GAL4-MEF2D, GAL4:LUC and CMV:Renilla, together with either wild-type HA-ERK5 (full length) or HA-ERK5 ΔTAD and EGFP-MEK5D or EGFP (control) as indicated. Four hours post transfection, cells were treated with either DMSO (control) or compound **26** (**a**), compound **25** (**b**), AX15836 (**c**) or BIX02189 (**e**) at concentrations indicated. Twenty-four post transfection, cells were lysed and firefly luciferase activity was measured and normalised to Renilla. The results are presented as the mean of three independent experiments ± SEM. Source data are provided as a Source Data file. **d** HEK293 cells were transfected as in (**a**). Twenty-four hours post transfection cells were lysed and processed as in **a**. **f** HEK293 cells were transfected with GAL4-MEF2D, GAL4:LUC and CMV:Renilla together with either wild-type HA-ERK5 (full length) or HA-ERK5 kinase dead and EGFP-MEK5D or EGFP (control) as indicated. Twenty-four hours post transfection cells were lysed and processed as in **a**. **g** and **h** HEK293 cells were transfected as in (**f**). Four hours post transfection, cells were treated with either DMSO (control) or compound **26** (**g**) or AX15836 (**h**) at concentrations indicated. Twenty-four hours post transfection cells were lysed and processed as in (**a**).

To measure the IC_50_ of ERK5i, transfected cells (as described above (Fig. 1c)) were incubated with increasing concentrations of inhibitors and luciferase activity measured. Compound **26** caused a dose-dependent decrease in MEK5D-driven luciferase activity; however the dose–response curve never reached zero, with 30–40% of maximal luciferase activity persisting, even at higher concentrations (Fig. 2a). This effect was also seen with compound **25** (an ERK5i similar to compound **26**) (Fig. 2b). Compound **26** also inhibits the bromodomain and extra terminal (BET) domain family member, BRD4, a protein that binds acetylated lysines and plays a role in gene regulation. To control for BRD4 being responsible for the residual activity in the ERK5:MEF2D assay we used AX15836 an ERK5i that has no BRD4 activity^28^. Strikingly AX15836 caused a paradoxical increase in ERK5:MEF2D-dependent luciferase expression over and above that seen with MEK5D (Fig. 2c).

We speculated that the residual activity that remained for compounds **25** and **26**, and the increase in activity with AX15836 was due to an ERK5-mediated induction of MEF2D activity in response to ERK5i binding. To test if ERK5i were able to drive ERK5:MEF2D activity in this assay we repeated these experiments but omitted MEK5D, so that ERK5 was un-phosphorylated and inactive (‘non-activated full length ERK5’). Indeed, compounds **26**, **25** and AX15836 all elicited a dose-dependent increase in ERK5:MEF2D luciferase activity. Moreover, for compounds **26** and **25** the level of induced activity was the same as the residual activity seen when MEK5D was present (Fig. 2a–c comparing ‘non-activated full length ERK5’ with ‘MEK5D-activated full length ERK5’).

The C-terminus of ERK5 contains a transcriptional transactivation domain (TAD)^6^ (Fig. 1a), which is known to interact with MEF2D^6^. To test if the C-terminus was responsible for the residual activity we produced a mutant of ERK5 (termed ERK5ΔTAD) that lacked the C-terminal extension, mimicking a naturally occurring ERK5 splice variant^42^ (Fig. 1a). MEK5D activated both full length ERK5 and ERK5ΔTAD equally in this system (Fig. 2d). Using MEK5D to activate ERK5ΔTAD we saw complete, saturable inhibition of ERK5:MEF2D-dependent luciferase activity with compounds **25**, **26** and AX15836 (Fig. 2a–c) consistent with their ERK5 kinase inhibitory activity. In agreement with the paradoxical activation of ERK5 being mediated by its C-terminal extension, there was no induction of luciferase activity when non-activated ERK5 ΔTAD (not activated by MEK5D) was incubated with either compounds **25**, **26** or AX15836 (Fig. 2a–c). Furthermore, using successive C-terminal deletion mutants we mapped the region responsible for compound **26**-induced transcriptional activation of ERK5:MEF2D to the previously reported minimal TAD (for more information see Supplementary Fig. 1).

The activation of ERK5:MEF2D seen with ERK5i was not seen when we used the MEK5 inhibitor, BIX02189; when tested with full length ERK5, BIX02189 fully inhibited ERK5:MEF2D luciferase activity with an IC_50_ of ~500 nM (Fig. 2e). To test if ERK5 kinase activity was required for paradoxical activation of ERK5 we repeated the same experiments with a kinase-dead mutant of ERK5 in which the catalytic aspartic acid was mutated to alanine (D182A), (Fig. 2f); both compounds **26** and AX15836 stimulated ERK5-dependent expression of luciferase by MEF2D with either wild type or kinase dead ERK5 (Fig. 2g, h). Phosphorylation of the ERK5 C-terminus and MEF2D promotes transcriptional activity. We therefore assessed C-terminal phosphorylation and MEF2D phosphorylation in the presence of MEK5D, compound **26** and AX15836. Although compounds **26** and AX15836 promoted phosphorylation of S754 in the ERK5 C-terminus, this was not required for ERK5i induced ERK5:MEF2D activity (Supplementary Fig. 2). Furthermore, we identified 18 MEK5-ERK5-driven phosphorylation sites on MEF2D, but phosphorylation of MEF2D was not induced by compound **26** or AX15836 (for more information see Supplementary Fig. 3 and Supplementary Table 1).

Taken together these results demonstrated that while compounds **25**, **26** or AX15836 are all very effective inhibitors of ERK5 kinase activity, they also promote paradoxical activation of the ERK5 C-terminal TAD. This effect does not require ERK5 kinase activity, ERK5 or MEF2D phosphorylation and is not shared with MEK5i (BIX02189), suggesting it is unique to those compounds that bind to ERK5.

### Generation of compound 26-resistant, kinase-active mutants of ERK5

In cell-based assays, compounds may exhibit off-target effects, which create false positives. These include: (i) activation of stress responses that may signal to ERK5; (ii) indirect effects on luciferase expression by affecting the transcriptional and translational machinery, or (iii) direct effects on luciferase activity. Therefore, it was critical to determine whether the transcriptional activation by ERK5i in the ERK5:MEF2D assay was a result of direct binding of ERK5i to the ERK5 kinase domain and not an off-target effect. We, therefore, set out to generate ERK5 mutants that lacked the ability to bind compound **26** but retained kinase activity.

**Fig. 3:**
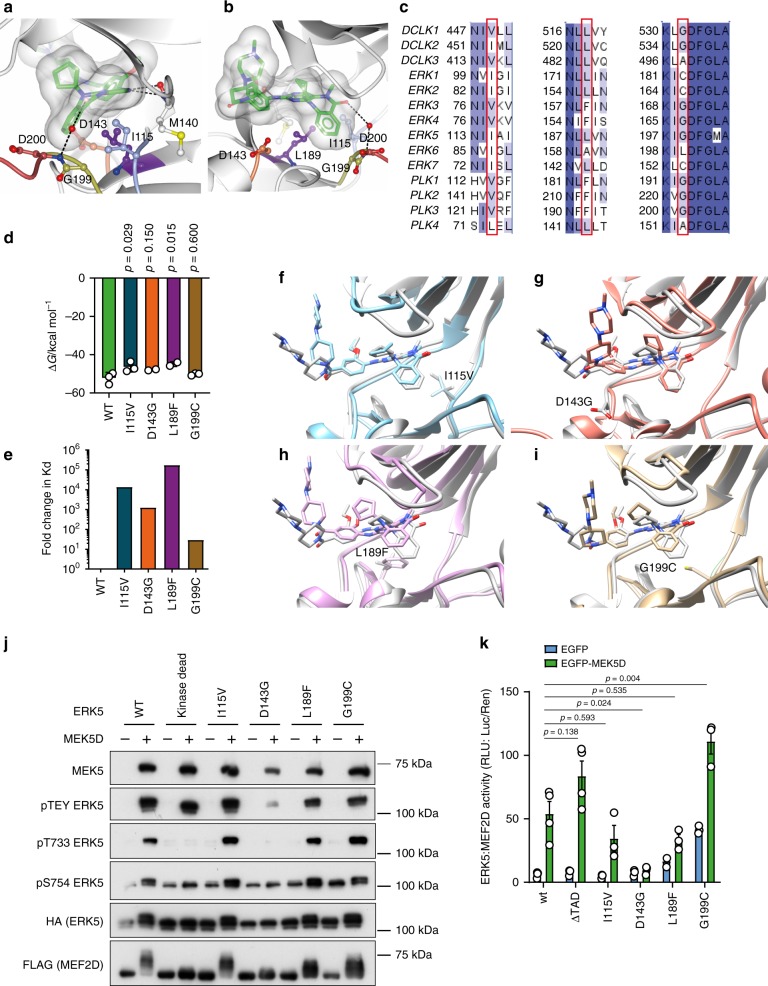
Generation of compound 26-resistant kinase-active mutants of ERK5. **a**, **b** Compound **25** (carbon atoms in green) bound to the ATP-binding pocket of ERK5 (PDB 4B99^37^). Side-chains of residues proposed by Elkins et al.^37^ as key to the selectivity and binding affinity of **25** for ERK5 are shown in ball-and-stick and coloured blue (Ile115), purple (Leu189), gold (Gly199) and coral (Asp143), respectively. Asp200 from the ‘DFG’ motif at the start of the activation-loop (red), and Met140 in the hinge region (grey) are also shown. A transparent molecular surface is drawn over compound **25**. Hydrogen bonds between **25** and ERK5 are shown as black dashed lines. The view in (**b**) is rotated ~90° about a vertical axis compared to the view in (**a**). Figure prepared using CCP4MG^84^. **c** Comparison of sequences across the DCLK, ERK and PLK families highlights conservation of Ile115, Leu189 and Gly199 (ERK5 numbering), respectively, as key to inhibition by compounds **25** and **26**. Sequences were retrieved from the UniProt database^85^, aligned using Clustal Omega^86^, and the alignment coloured by sequence identity (where darker blue indicates higher conservation) and annotated using Jalview^87^. **d** Calculated binding energies for compound **26** to wild-type ERK5, and the mutant variants I115V, D143G, L189F and G199C, as derived from molecular dynamics simulations and MM/PBSA calculation (unadjusted for entropic contribution); the reduction in binding energy is statistically significant by one-way ANOVA for binding of compound **26** to mutants I115V (*p* = 0.0282) and L189F (*p* = 0.0149) when compared to binding to wild-type ERK5. Source data are provided as a Source Data file. **e** Estimated log-fold change in Kd derived from calculated binding energies demonstrating notably weaker affinity of compound **26** for all mutant variants of ERK5 compared to wild-type. Source data are provided as a Source Data file. **f**–**i** Comparison of compound **26** binding to ERK5 wild-type (grey) and I115V (**f**, blue), D143G (**g**, coral), L189F (**h**, purple) or G199C (**i**, gold) in silico, derived from molecular dynamics simulations with wild-type and mutated residue shown as sticks to denote mutation site. The D143G mutation causes compound **26** to shift toward the P-loop. The I115V, L189F and G199C mutations cause compound **26** to shift out into a shallower binding position in the active site, resulting in the weaker binding energies observed. Figures were prepared using UCSF Chimera. **j** HEK293 cells were transfected with FLAG-MEF2D and either wild-type HA-ERK5, kinase dead HA-ERK5, HA-I115V ERK5, HA-D143G ERK5, HA-L189F ERK5 or HA-G199C ERK5, and either EGFP-MEK5D or EGFP. Twenty-four hours post transfection cells were lysed, subjected to SDS-PAGE and immuno-blotted with the antibodies shown. The experiment was repeated three times and a representative image is shown. **k** HEK293 cells were transfected with GAL4-MEF2D, GAL4:LUC and CMV:Renilla, together with either wild-type HA-ERK5, HA-ERK5 ΔTAD, HA-I115V ERK5, HA-D143G ERK5, HA-L189F ERK5 or HA-G199C ERK5 and either EGFP-MEK5D or EGFP. Twenty-four hours post transfection, cells were lysed and firefly luciferase activity was measured and normalised to Renilla. The results are presented as the mean of at least three independent experiments ± SEM. Source data are provided as a Source Data file.

The X-ray crystal structure of the kinase domain of ERK5 in complex with compound **25**^37^ and pan-kinase binding selectivity data for the highly similar compound **26**^36^ suggested that the presence of a small residue immediately prior to the DFG motif, at the start of the activation-loop (G199 in ERK5), combined with a leucine in the base of the ATP-binding pocket (L189 in ERK5), was important to accommodate the cyclopentyl substituent of compounds **25** and **26** (Fig. 3a, b). This combination of residues, although common among kinases more generally, is rare in members of the MAPK family, being found only in ERK5 (Fig. 3c). ERK1, ERK2, ERK7 and NLK, all have a cysteine at the position equivalent to ERK5 G199 combined with a leucine at the position equivalent to ERK5 L189, and these kinases are not inhibited by compound **26**. By contrast, ERK3 and ERK4, and the polo-like kinases PLK1, PLK2 and PLK3, which are also not inhibited by compound **26**, all have glycine at the position equivalent to G199 in ERK5, while the residue equivalent to ERK5 L189 is the bulkier phenylalanine. We hypothesised therefore that introducing individual mutations in the ATP-binding site of ERK5 of L189 to phenylalanine (L189F) and G199 to cysteine (G199C) would generate mutant ERK5 variants resistant to inhibition by compound **26**, while retaining kinase activity. Elkins et al.^37^ postulate that binding affinity and selectivity are also driven by hydrophobic interaction of I115 at the back of the ATP-binding pocket with the *N*-methyl substituent of compounds **25** and **26**. Comparison of binding data and sequence conservation across the family of DCLKs suggested that replacement of I115 with valine (as observed in DCLK1 and 3) would negatively impact binding of compounds **25** and **26** (Fig. 3a–c), again generating a kinase-active but inhibitor-resistant mutant. Finally, the presence of aspartate (D143 in ERK5) at the mouth of the ATP-binding pocket just C-terminal to the hinge region is proposed to contribute to tight binding of compounds **25** and **26** by securing the hinge-binding moiety in place^37^ (Fig. 3a, b). The side-chain of D143 forms a helix cap, stabilising the short αD helix, which follows the hinge region (Supplementary Fig. 4A). Structure-guided sequence alignments across the kinome show that this residue is often an amino acid such as aspartate, asparagine, glutamate or serine, which can form a helix cap (Supplementary Fig. 4B)^43^; truncation to glycine (D143G) removes both the steric constraints imposed on compound binding and the αD helix cap.

We undertook molecular dynamics simulations of compound **26** bound to wild-type ERK5, as well as mutants I115V, D143G, L189F and G199C to predict relative binding energies by the MM/PBSA method (Fig. 3d). We noted no significant changes in the flexibility of either the ligand or the binding site (Supplementary Fig. S4C, D) over the short simulation time; however, as anticipated, we observed unfavourable changes to the binding energies of all mutants, with statistically significant reductions for mutants I115V and L189F compared to wild-type. Converting the differences in binding energy to estimate a log-fold change in binding affinity (Fig. 3e), predicted notably weaker binding of compound **26** to all the ERK5 mutants simulated, with the binding position of compound **26** compromised by all mutations (Fig. 3f–i).

We generated these mutants and expressed them in the presence and absence of MEK5D. MEK5D was able to phosphorylate all of the mutants on the TEY motif, albeit D143G to a lesser extent than the others (Fig. 3j). An ERK5 band-shift was observed for wild type, I115V, L189F and G199C but not kinase dead or D143G, suggesting that D143G lacks kinase activity. Consistent with this, phosphorylation of T733, a known autophosphorylation site was detected on wild type, I115V, L189F and G199C but not kinase dead or D143G. Furthermore, S754 phosphorylation (another ERK5 autophosphorylation site) was increased following MEK5D expression on wild type, I115V, L189F and G199C, but not kinase dead or D143G. Interestingly, G199C had higher basal S754 phosphorylation than wild type (Fig. 3j). As we saw a MEF2D band-shift when ERK5 was activated with MEK5D in Supplementary Fig. 3C, we used this as a readout of ERK5 kinase activity in cells; this demonstrated that wild type, I115V, L189F and G199C were all active MEF2D kinases, whereas D143G was not (Fig. 3j). Next, we assessed the activity of these mutant forms of ERK5 in the ERK5:MEF2D luciferase assay; L189F and G199C had a higher basal activity compared to wild type and I115V, L189F and G199C were all activated by MEK5D, whereas D143G was not (Fig. 3k). Thus, we were able to generate three kinase-active ERK5 mutants with predicted reduced binding ability for compound **26**.

### Binding of compounds 26 or AX15836 to the ERK5 kinase domain is required for paradoxical ERK5:MEF2D transcriptional activation

**Fig. 4:**
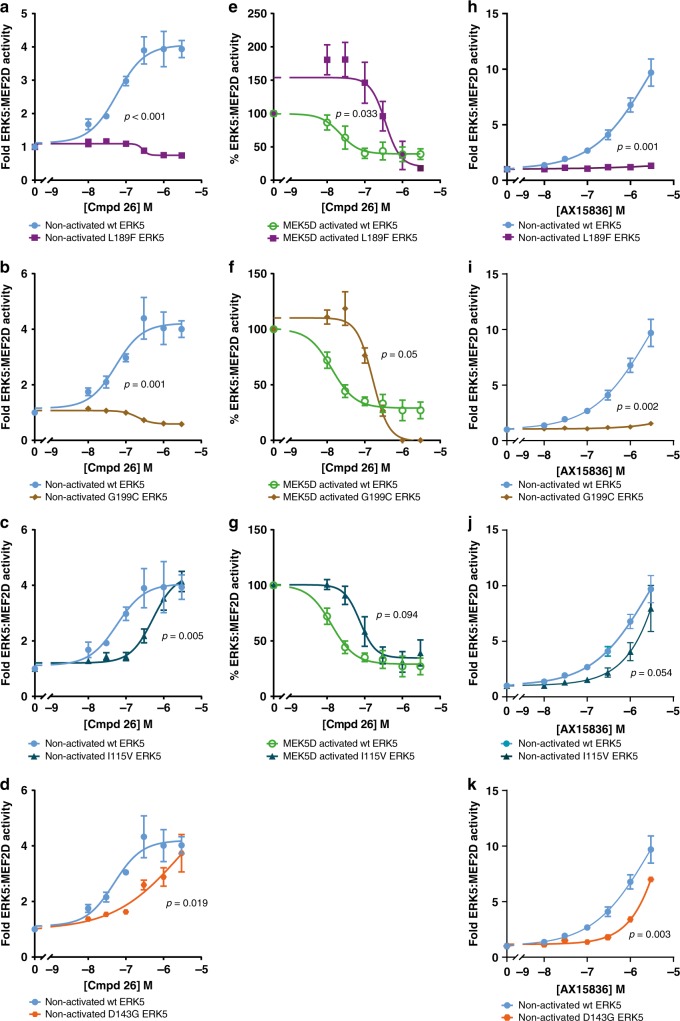
Binding of compound 26 and AX15836 to the ERK5 kinase domain is required for paradoxical ERK5:MEF2D transcriptional activation. **a**–**d** and **h**–**k** HEK293 cells were transfected with GAL4-MEF2D, GAL4:LUC and CMV:Renilla together with wild-type HA-ERK5 (full length) and either HA-L189F ERK5 (**a**, **h**), HA-G199C ERK5 (**b**, **i**), HA-I115V ERK5 (**c**, **j**) or HA-D143G ERK5 (**d**, **k**) and EGFP then 4 h post transfection treated with compound **26** (**a**–**d**) or AX15836 (**h**–**k**) at the concentrations indicated. Twenty-four hours post transfection, cells were lysed and firefly luciferase activity was measured and normalised to Renilla. The results are presented as the mean of three experiments ± SEM. Source data are provided as a Source Data file. **e**–**g** HEK293 cells were transfected with GAL4-MEF2D, GAL4:LUC and CMV:Renilla together with wild-type HA-ERK5 (full length) and either HA-L189F ERK5 (**e**), HA-G199C ERK5 (**f**) or HA-I115V ERK5 (**g**) and EGFP-MEK5D then 4 h post transfection treated with compound **26** at the concentrations indicated. Twenty-four hours post transfection, cells were lysed and processed as in (**a**–**d** and **h**–**k**).

We conducted dose–response experiments comparing the predicted compound **26**-resistant ERK5 mutants with wild-type ERK5 in the ERK5:MEF2D assay. With non-activated ERK5, L189F and G199C were both completely resistant to compound **26** with no induction of ERK5:MEF2D (Fig. 4a, b), while I115V and D143G had reduced sensitivity to compound **26** compared to wild type so that the induction curve shifted to the right (Fig. 4c, d). For MEK5D-activated ERK5 we assessed the effects on L189F, G199C and I115V, but not D143G (as it could not be activated). L189F, G199C and I115V ERK5 mutants each showed a reduction in sensitivity to compound **26**, with the effect more notable for L189F and G199C compared to I115V (Fig. 4e–g). This is consistent with the induction of non-activated ERK5:MEF2D transcriptional activity for these mutants.

AX15836 is a derivative of XMD8-92 and compound **26**, so we speculated that the compound **26** drug-binding mutants would also be resistant to AX15836. We therefore repeated the dose–response experiments comparing the compound **26**-resistant ERK5 mutants to wild type in the ERK5:MEF2D assay. With non-activated ERK5, L189F and G199C were both completely resistant to AX15836 (Fig. 4h–i) and I115V and D143G had a reduced effect (Fig. 4j–k), comparable to compound **26**.

These results confirmed the structural predictions that I115V, D143G, L189F and G199C would reduce binding of compound **26** to ERK5. These mutants fell into two groups; those on which compound **26** had little or no effect (L189F and G199C), and those on which compound **26** had a reduced effect (I115V and D143G). These mutants exhibited similar responses to AX15836. Significantly, these results showed that direct binding of compound **26** or AX15836 to the ERK5 kinase domain was required for the paradoxical drug-induced activation of ERK5:MEF2D transcriptional activity, confirming that compound **26** or AX15836 were acting ‘on target’ to activate the ERK5 TAD.

### Compound 26 and AX15836 promote ERK5 nuclear localisation

**Fig. 5:**
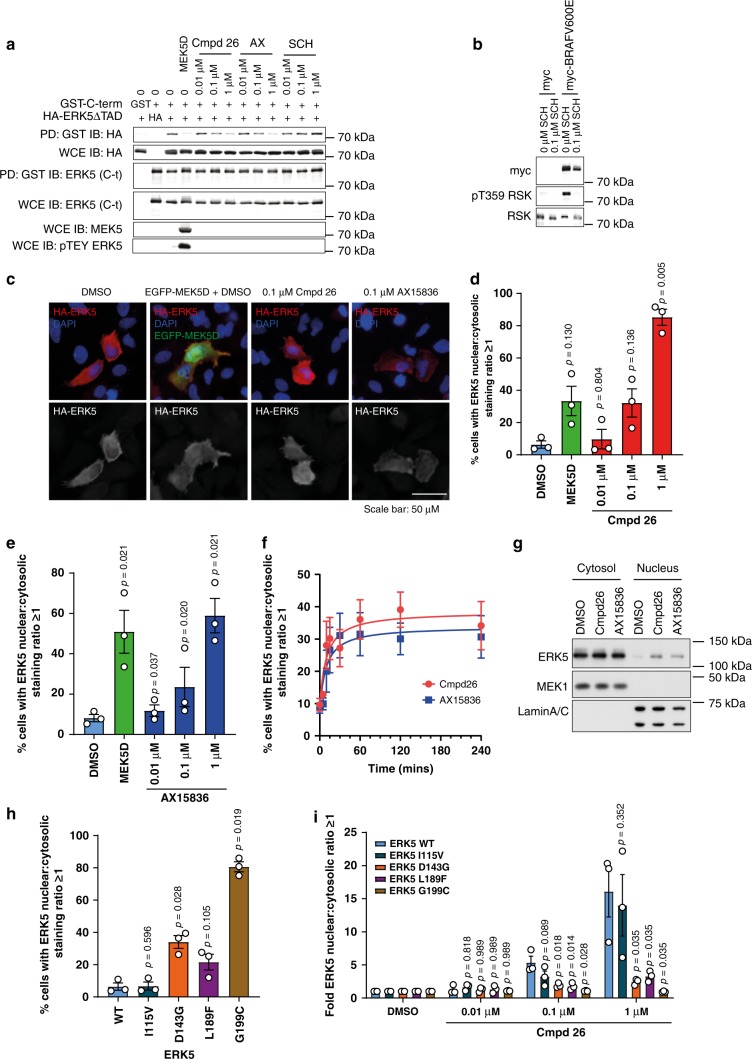
Compound 26 and AX15836 promote ERK5 nuclear localisation. **a** HEK293 cells were transfected with GST-ERK5 C-term, HA-ERK5 ΔTAD, GST, HA, EGFP-MEK5D or GFP as indicated. Four hours post transfection, cells were treated with compound **26**, AX15836 (AX), SCH772984 (SCH) or DMSO as control (0) at the concentrations indicated for 24 h then the cells were lysed. Lysates were incubated with glutathione sepharose to precipitate GST-ERK5 C-term and measure co-pulldown of HA-ERK5 ΔTAD. Pulldowns (PD) were immuno-blotted (IB) for GST-ERK5 C-term using ERK5 (C-terminal) antibodies and HA-ERK5 ΔTAD using HA antibodies. To measure expression, whole-cell extracts (WCE) were blotted using ERK5 (C-terminal), HA and MEK5. Phosphorylation of ERK5 by MEK5D was measured using phospho-ERK5 TEY antibodies. Source data are provided as a Source Data file. **b** HEK293 cells were transfected with either myc-tag or myc-BRAF^V600E^ as indicated. Four hours post transfection, cells were treated with 0.1 µM SCH772984 (SCH) or DMSO as control (0). Cells were harvested and immuno-blotted for myc (for expression of myc-BRAF^V600E^), phospho-T359 RSK and total RSK. A representative image of three independent experiments is shown. Source data are provided as a Source Data file. **c** HeLa cells were transfected with wild-type HA-ERK5 and either EGFP or EGFP-MEK5D. Twenty-four hours post transfection, cells were treated with compound **26**, AX15836 or DMSO as indicated. Thirty minutes after treatment cells were fixed, permeabilised, blocked and stained with anti-HA then donkey-anti-mouse 568 Alexafluor and DAPI. Images were captured by high-content fluorescence microscopy. **d** and **e** HeLa cells were transfected with wild-type HA-ERK5 and either EGFP or EGFP-MEK5D. Four hours post transfection, cells were treated with compound **26** (**d**), AX15836 (**e**) or DMSO as indicated. Twenty-four hours post transfection, cells were fixed, permeabilised, blocked and stained with anti-HA then donkey-anti-mouse 568 Alexafluor and DAPI. High-content microscopy was used to determine the levels of nuclear and cytosolic HA-ERK5 staining. Results are presented as the % of transfected cells with nuclear≥cytosolic HA staining of three independent experiments ± SEM. Source data are provided as a Source Data file. **f** HeLa cells were transfected as in (**d**) and (**e**) then treated with compound **26** or AX15836 for the times indicated. Cells were then treated and processed as in (**d** and **e**). **g** HeLa cells were treated with 0.1 µM compound **26**, AX15836 or DMSO for 30 min. Cells were harvested and the cytosolic and nuclear fractions were isolated and immuno-blotted for ERK5, MEK1 (cytosolic marker) and lamin A/C (nuclear marker). A representative image of three independent experiments is shown. Source data are provided as a Source Data file. **h** HeLa cells were transfected with wild-type HA-ERK5, HA-I115V ERK5, HA-D143G ERK5, HA-L189F ERK5 or HA-G199C ERK5 and EGFP. Twenty-four hours post transfection, cells were treated and processed as in (**d**). **i** HeLa cells were transfected with wild-type HA-ERK5, HA-I115V ERK5, HA-D143G ERK5, HA-L189F ERK5 or HA-G199C ERK5 and EGFP. Four hours post transfection, cells were treated with compound **26** or DMSO as indicated. Twenty-four post transfection, cells were treated and processed as in (**d**).

In its inactive state, ERK5 is predominantly cytosolic and translocates into the nucleus upon activation by MEK5^12^. This is regulated by an intramolecular interaction between the N-terminal kinase domain and the C-terminus, which ensures mutual repression of the kinase and TAD functions; this is de-repressed by MEK5-catalysed phosphorylation of ERK5, which activates the kinase domain and exposes the NLS and TAD^11,12^. In order to determine if compound **26** or AX15836 mimic MEK5D by disrupting this interaction, we co-expressed the HA-ERK5 ΔTAD mutant (i.e., just the kinase domain) with GST-ERK5C-term. Pulldown with glutathione Sepharose beads showed that the HA-ERK5ΔTAD associated with the GST-ERK5C-term; furthermore, expression of MEK5D to drive TEY phosphorylation of HA-ERK5ΔTAD caused it to dissociate from GST-ERK5C-term (Fig. 5b). Compound **26** and AX15836 both caused a dose-dependent dissociation of HA-ERK5ΔTAD from the GST-ERK5C-term despite failing to promote TEY phosphorylation. As a control we used an ERK1/2 inhibitor, SCH772984^44^, which is inactive towards ERK5. SCH772984 had no effect demonstrating that it was an ERK5i selective effect (Fig. 5a). To show that SCH772984 was active in cells, we expressed myc-BRAF^V600E^ to stimulate ERK1/2 activity and measured phosphorylation of T359 on RSK, an ERK1/2 phosphorylation site. SCH772984 was active as it blocked this phosphorylation (Fig. 5b).

Prompted by this we assessed whether compound **26** or AX15836 treatment led to the exposure of the NLS to promote ERK5 nuclear translocation. We expressed HA-ERK5 in HeLa cells and imaged HA-ERK5 localisation using fluorescence microscopy. In untreated cells the majority of HA-ERK5 was seen in the cytosol. As a positive control, we co-expressed EGFP-MEK5D, which increased HA-ERK5 nuclear staining. Next, we assessed the effects of compound **26** or AX15836 and also observed an increase in HA-ERK5 nuclear staining (Fig. 5c). We used high-content fluorescence microscopy to score the number of cells with a higher nuclear to cytosolic staining for HA-ERK5. Using this method, we again observed that co-expression of MEK5D increased HA-ERK5 nuclear translocation, as expected. We also demonstrated that compound **26** or AX15836 promoted HA-ERK5 nuclear translocation in a dose-dependent manner (Fig. 5d, e). An increase in HA-ERK5 nuclear localisation in response to compound **26** or AX15836 was seen after 15 min, plateaued at 1 h (Fig. 5f) and was sustained for up to 24 h (Fig. 5d, e). Consistent with these experiments we also saw an increase in endogenous ERK5 in the nucleus following treatment of HeLa cells with compound **26** or AX15836 (Fig. 5g).

We extended these experiments to determine the cellular localisation of the compound **26**-resistant ERK5 mutants and the effects of compound **26** on these mutants. For the drug-resistant mutants, I115V staining was mostly cytosolic, similar to wild-type ERK5. D143G and L189F exhibited a higher basal nuclear staining than wild type. G199C had an increased nuclear staining with 80% of cells having more G199C staining in the nucleus than in the cytosol (Fig. 5h). These results reflected the basal ERK5:MEF2D activity measured in Fig. 3k (with the exception of D143G, which had a basal ERK5:MEF2D activity that was comparable to wild-type ERK5). The compound **26**-resistant mutants had an attenuated nuclear localisation in response to compound **26** treatment. For I115V the effect of compound **26** was slightly reduced at 0.1 µM but exhibited a similar effect to wild type at 1 µM (Fig. 5i), as was seen with ERK5:MEF2D activity in Fig. 4c. D143G, L189F and G199C localisation was resistant to compound **26** treatment (Fig. 5i), consistent with the attenuated effect of compound **26** on the ERK5:MEF2D activity of these mutants shown in Fig. 4.

These results not only showed that compound **26** or AX15836 cause exposure of the NLS and TAD, driving nuclear localisation of ERK5, but also that the level of nuclear localisation of ERK5 correlated with transcriptional activity (comparing the nuclear localisation of the ERK5 compound **26**-resistant mutants with basal ERK5:MEF2D activity), and provided further evidence that the compound **26**-resistant mutants were less responsive to compound **26**.

### Compound 26 and AX15836 activate expression from the KLF2 promoter

**Fig. 6:**
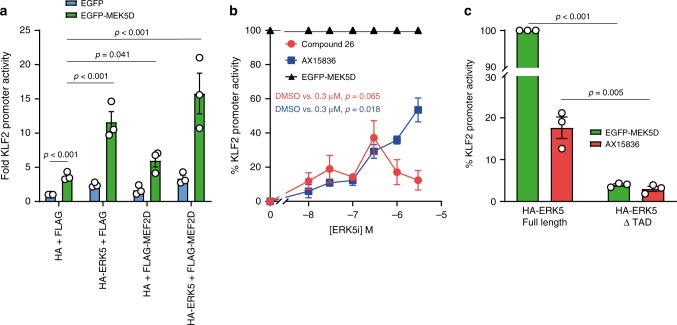
ERK5i induce expression from the KLF2 promoter and is dependent on ERK5 TAD. **a** HEK293 cells were transfected with KLF2:LUC and CMV:Renilla together with either FLAG or FLAG-MEF2D and either HA or HA-ERK5 (full length), and either EGFP (control) or EGFP-MEK5D as indicated. Twenty-four hours post transfection, cells were lysed and firefly luciferase activity was measured and normalised to Renilla. The results are presented as the mean of three independent experiments ± SEM. Source data are provided as a Source Data file. **b** HEK293 cells were transfected with KLF2:LUC and CMV:Renilla together with FLAG and HA, with either EGFP or EGFP-MEK5D (to activate endogenous ERK5). Four hours post transfection, cells transfected with KLF2:LUC and CMV:Renilla together with FLAG, HA and EGFP were treated with either compound **26**, AX15836 or DMSO (control) at the concentrations indicated. Cells transfected with KLF2:LUC and CMV:Renilla together with FLAG, HA and EGFP-MEK5D were treated with DMSO. Twenty-four hours post transfection, cells were lysed and firefly luciferase activity was measured and normalised to Renilla. The results are presented as % KLF2 promoter activity where MEK5D-driven (through endogenous ERK5) KLF2 promoter activity is 100% (mean of three independent experiments ± SEM). Source data are provided as a Source Data file. **c** HEK293 cells were transfected with KLF2:LUC and CMV:Renilla, together with FLAG-MEF2D and either HA-ERK5 (full length) or HA-ERK5 ΔTAD, and either EGFP (control) or EGFP-MEK5D. Four hours post transfection cells were treated with either 300 nM AX15836 or DMSO (control). Twenty-four hours post transfection cells were lysed and processed as in **b**.

Finally, we wanted to assess the effects of ERK5i on an ERK5-regulated promoter. To this end, we used KLF2 that is known to be regulated by ERK5 and MEF2D^44^. We expressed a luciferase reporter regulated by −924/+14 bp of the 5′UTR of the *KLF2* gene. Co-expression of EGFP-MEK5D was able to increase KLF2 reporter activity 3.5-fold; acting through endogenous ERK5 and MEF2D. Expression of MEF2D and ERK5 increased KLF2 reporter activity 15-fold in the presence of MEK5D confirming their role in regulating the KLF2 promoter (Fig. 6a). We used this system to measure the effects of compound **26** or AX15836 on endogenous ERK5. Both of these ERK5i increased KLF2 promoter activity in a dose-dependent manner; however, the dose-dependency for each compound was different. The effect of compound **26** peaked at 300 nM before reducing at higher concentrations, whereas the induction observed with AX15836 increased as the concentration of drug increased (Fig. 6b). To test if the bi-phasic effect of compound **26** was due to the induction of S754 phosphorylation (Supplementary Fig. 2), we repeated this experiment comparing the effects of over-expressed wt ERK5 with the ERK5 S754A mutant, but both wt and the S754A mutant were inhibited at higher concentrations of compound **26** (Supplementary Fig. 5). However, as compound **26** is known to inhibit BRD4, we tested to see if KLF2-luciferase reporter activity was sensitive to BRD4 inhibition using JQ1. At 0.1 µM JQ1 KLF2 reporter activity was inhibited by ~30% (Supplementary Fig. 6). This suggests that at higher concentrations of compound **26** (>1 µM) the reduction in KLF2-luciferase reporter activity may be due to BRD4 inhibition. Notably, AX15836, which does not inhibit BRD4^28^, did not cause inhibition at high doses (Fig. 6b).

We then tested the role of the C-terminus of ERK5 using the KLF2 reporter system, with MEK5D as a positive control. Here, 300 nM AX15836 increased full length ERK5 driven KLF2 reporter activity and this was dependent on the C-terminus of ERK5 as the ERK5ΔTAD was unable to support KLF2 promoter activity in the presence of AX15836 or MEK5D (Fig. 6c). These results demonstrate that ERK5i are able to drive paradoxical transcriptional activation of a canonical ERK5-regulated gene, as well as the ERK5:MEF2D system.

## Discussion

The identification of ERK5 as a potential anti-cancer and anti-inflammatory drug target has seen academic groups and pharmaceutical companies invest in developing small-molecule inhibitors of the ERK5 kinase domain (ERK5i). Kinases are attractive, ‘druggable’ targets due to their active site lying in a deep cleft between the canonical N- and C-terminal lobes and by 2015, 28 kinase inhibitors had received US FDA approval^45^. However, intensive research and clinical experience have identified two main problems that affect the efficacy of kinase inhibitors: (i) innate or acquired resistance to the kinase inhibitor due to mutation of the intended target^46^, mutation of other pathway components or pathway remodelling^47^ and (ii) unintended activation of the target pathway^48^, either by inhibition of negative feedback pathways^49^ or through inhibitor binding to the kinase resulting in paradoxical activation (the latter termed ‘inhibitor hijacking of kinase activation’ by Okuzumi et al*.*^50^).

Our results show that ERK5i bind directly to the ERK5 kinase-active site and subsequently promote a conformational change in the kinase domain that dissociates the NLS and transcriptional activation domain (TAD), driving nuclear translocation of ERK5 and stimulation of a known ERK5-regulated promoter (KLF2). Therefore, ERK5i binding causes paradoxical activation of the ERK5 TAD and its downstream targets. Use of ERK5i that activate the ERK5 TAD and ERK5-dependent pathways may not only hinder research into defining the role of ERK5 in cells but may also impede the therapeutic potential of ERK5i. To aid the development of ERK5 inhibitors as potential therapeutics and tool compounds we set out to understand how ERK5i activated ERK5.

Several lines of evidence indicate that ERK5i elicit paradoxical TAD activation by binding directly to the kinase-active site rather than through off-target effects or feedback loops. Multiple feedback loops function in the ERK1/2 pathway (reviewed in Caunt et al.)^51^ and although none have been described to date for the ERK5 pathway they are likely to exist. We therefore set out to address if inhibition of ERK5 by ERK5i repressed a negative feedback loop, leading to activation of a non-inhibitor bound pool of ERK5 that could lead to pathway output. First, we used kinase dead ERK5. Had ERK5 kinase activity been required for paradoxical pathway activation then kinase dead ERK5 would be insensitive to ERK5i. However, both compound **26** or AX15836 ‘activated’ kinase dead ERK5 in the ERK5:MEF2D assay (Fig. 2). In addition, we did not see an increase in phosphorylation of the TEY motif in the ERK5 activation-loop with compound **26** or phosphorylation of the ERK5 substrate MEF2D showing that neither MEK5 nor ERK5 kinase activity was activated in the presence of ERK5i (Supplementary Figs. 2 and 3). Further evidence against a negative feedback loop came from the MEK5 inhibitor, BIX02189. If inhibition of ERK5 blocked a feedback loop to reactivate the pathway then MEK5 inhibition would be expected to do the same; indeed, this is seen with MEK1/2 inhibitors in cells with wild-type BRAF^51^. In fact, BIX02189 fully inhibited ERK5:MEF2D activity; no residual activity or pathway activation was seen (Fig. 2). Taken together, these results argue against a feedback mechanism being responsible for increased output of ERK5 activity in the presence of ERK5i. To rule out any off-target mechanisms we generated ERK5i-insensitive, kinase-active mutants of ERK5 (Fig. 3). These fell into two classes with attenuated sensitivity to compound **26**; mutations that reduced the effect of compound **26** (I115V and D143G) and those that blocked the effect of compound **26** (L189F and G199C) on ERK5:MEF2D transcriptional activity (Fig. 4). These mutations also blocked ERK5 nuclear localisation in response to compound **26** (Fig. 5). Taken together, these results show that it is the direct binding of the inhibitors (compound **26** or AX15836) to the ERK5 kinase-active site that promoted paradoxical activation.

How does binding of ERK5i to the kinase domain activate ERK5 transcriptional activity? The ERK5 kinase domain and TAD are known to reciprocally repress each other in cells^6,11^. When ERK5 is activated by MEK5 this leads to autophosphorylation of the ERK5 C-terminus, which promotes transcriptional activity^11^. It is known that inhibitor binding can alter the dynamic ensemble of kinase conformations to stabilise a specific conformation^52^. The crystal structure of ERK5 in complex with compound **25** shows that ERK5 is in an ‘active’ conformation using PKA in complex with ATP and PKI as a reference (Supplementary Fig. 7). Interestingly, comparison of the crystal structures of ERK5 in complex with compound **25** (PDB 4B99) and with ATP and the PB1 domain of MEK5 (PDB 4IC7) (Supplementary Fig. 7) reveals differences in the conformation of the activation-loop and the orientation of the αC-helix that might propagate to changes in the C-terminal portion of the protein (however, both of these regions are also involved in crystal lattice contacts so caution should be exercised in the interpretation of the observed differences). To assess the potential impact of AX15836 binding to ERK5 we conducted molecular dynamic simulations. The binding energy was less favourable for AX15836 compared to compound **26**, however, there was a reduction in the flexibility of the activation-loop most notably at positions 215 and 216—adjacent to the TEY motif at 218–220 (Supplementary Fig. 8). These observations suggest that either the conformational changes in the kinase domain are involved in interactions between the kinase domain and C-terminus, or that the inhibitor directly blocks such interactions. Further molecular analysis is required to characterise these intramolecular interactions to fully validate this model.

Paradoxical activation of ERK5 is the second major hurdle that ERK5 inhibitor programmes have encountered. The first hurdle emerged from studies with the founding ERK5 inhibitor, XMD8-92. This compound is tenfold more selective for ERK5 over other kinases tested and phenocopied siRNA knockdown of ERK5^18,27,33,53–56^. Subsequently, Lin et al.^28^ developed AX15836, a next generation ERK5i with high selectivity and increased potency for ERK5. However, XMD8-92 and AX15836 were found to diverge in key cellular activities, leading to the suggestion that XMD8-92 had ERK5-independent off-target effects. Indeed, XMD8-92 was found to bind to BRD4^28^. However, Lin et al.^28^ also noted a discrepancy between the effects of knockdown of ERK5 and ERK5 inhibition by AX15836; knockdown of ERK5 prevented IL-6 and IL-8 production from HUVEC and BEAS-2B cells, but AX15836 did not. Our results now provide a possible explanation for why AX15836 was unable to phenocopy ERK5 knockdown; namely because AX15836 paradoxically activates ERK5 transcriptional activity. Further studies are now required to address the role of transcriptional regulation by ERK5 in this and other models.

These observations raise two critical questions moving forward. First, since ERK5 kinase inhibitors can activate the ERK5 TAD, which of these two activities (kinase domain or TAD) is critical for the biological effects of ERK5 in diseases such as cancer and inflammation? In this context it is notable that naturally occurring ERK5 splice variants exist that lack either the C-terminal TAD or the N-terminus, including the kinase N-lobe (such that the kinase will be inactive)^42,57^; it will be interesting to see if the embryonic lethality associated with ERK5 knockout is replicated by a ∆TAD deletion or a catalytically inactive kinase mutant. The second critical question is whether paradoxical activation by ERK5i can be engineered out? The answer to this question is likely to be yes. The relative magnitude of effect of compound **26** and AX15836 (Fig. 2) suggests there is scope for this effect to be eliminated through the combination of structure-guided drug design and cell-based screening. Using BRAF as an example, success has been seen with the paradox-breaking compounds, PLX7904 and PLX8394. These inhibitors suppress the growth of melanoma cells with BRAF^V600E^ but, unlike vemurafenib, do not activate ERK1/2 signalling in cells harbouring mutant KRAS or in cells that have activated tyrosine kinases^58^.

To date, two mechanisms of paradoxical pathway activation have been described for kinase inhibitors. The first is by priming kinases for activation. For example, selective inhibitors of PKB promote its membrane localisation, phosphorylation of its regulatory sites (T308 and S473) by the upstream kinases, PDK1 and mTOR, respectively^52^, and acquisition of a phosphatase-resistant conformation^59^. As a result, when the inhibitor dissociates, PKB is catalytically active and primed for activity^50^. Similar observations have been made for PKC^60^, PKD^61^ and AMPK^62^. The second way small-molecule kinase inhibitors can paradoxically activate a pathway is by transactivation of non-drug-bound isoforms in homo- or hetero-dimer pairs. The most notable example are the RAF inhibitors such as vemurafenib. This inhibitor was developed to target the BRAF^V600E^ mutant and shows potent anti-tumour effects in tumours carrying this mutation. Critically, while vemurafenib can inhibit BRAF^V600E^, which signals as a monomer, it can activate wild-type RAF kinases that act as RAS-dependent CRAF-CRAF homodimers or BRAF-CRAF heterodimers. In this case, the drug-bound protomer trans-activates the drug-free protomer, leading to pathway activation. Inhibitor binding also promoted membrane localisation of RAF and interaction with RAS-GTP^63,64^. Similar observations have been made for bosutinib binding to the pseudokinase HER3^65,66^ to induce an EGFR-dependent proliferative signal^67^. What we observe with inhibitor binding to ERK5 presents a third and entirely new way that kinase inhibitors can cause paradoxical activation of their intended target. Direct binding of the inhibitor to the kinase causes intramolecular changes that cause activation of the target molecule. This is neither dependent on priming phosphorylation and inhibitor dissociation, nor transactivation of a dimer partner to induce phosphorylation of downstream targets. We term this ‘direct paradoxical activation by kinase inhibitors’.

In conclusion, we show that ERK5i paradoxically activate ERK5 transcriptional activity by binding to the ERK5 catalytic site and de-repressing the C-terminal TAD function; this represents a third model whereby kinase inhibitors paradoxically activate their intended target. This may explain why the most up-to-date ERK5i that lack BRD4 activity do not phenocopy ERK5 small-interfering RNA (siRNA) knockdown. As a result, caution should be exercised when interpreting cellular data using compound **26** (ERK5-IN-1/XMD17-109) or AX15836. Furthermore, paradox-breaking ERK5i should be identified to progress our understanding of ERK5 structure-function, ERK5 biology and to inform ERK5 drug discovery programmes.

## Materials and methods

### Materials

Cell culture reagents were purchased from Invitrogen and Sigma. Compounds **25** and **26** (ERK5-IN-1) were originally provided by Professor Nathanael Gray and Dr. Jinhua Wang, Department of Biological Chemistry and Molecular Pharmacology, Harvard Medical School, Boston, USA then latterly compound **26** was purchased from Selleckchem. AX15836 and JQ1 are from Tocris. BIX02189 is from Selleckchem. Anti-HA antibody for immunoblots was provided by the Babraham Institute Monoclonal Facility^40,41^ and anti-HA (12CA5) antibody for immuno-fluorescence^68^ was from Roche/Sigma. Phospho-ERK5 TEY (3371) antibody was from Cell Signaling Technology^40,41^. Phospho-T733 and phospho-S754 ERK5 antibodies were a kind gift from Dr. Atanasio Pandiella, University of Salamanca, Spain^16^. Anti-Flag antibody (M2) is from Sigma^69^. Anti-MEK5 antibody (AB3184) is from EMD Millipore^22^, anti-lamin A/C (636)^70^, ERK5 (C-7)^26^ and myc (9E10)^71^ antibodies are from Santa Cruz and anti-MEK1/2^72^, phospho-T359 RSK^73^ and total RSK^74^ from Cell Signaling Technology. Secondary antibodies were from BioRad^40,41^.

## Methods

### Cell culture

HEK293 and HeLa were cultured in Dulbecco's modified Eagle medium (DMEM) supplemented with 10% FBS at 37 °C in an atmosphere of 5% CO_2_ and 95% humidity.

### Plasmids

The HA-ERK5 and EGFP-MEK5D constructs have been described previously and were derived from constructs provided by J.D. Lee. GAL4-MEF2D (GAL4 DNA binding domain fused to a.a. 87–428 of rat MEF2D) was from J.C.McDermott, Centre for Research in Biomolecular Interactions, York University, Toronto, Canada; GAL4:LUC reporter from D. Gillespie, University of Glasgow, Glasgow, UK; GST-ERK5 C-term (aa 401–815) was from J. Lizcano, Universitat Autònoma de Barcelona, Spain. A luciferase reporter gene driven by the KLF2 promoter (−924 + 14) was a kind gift from J. Abe, Department of Cardiology, Division of Internal Medicine, The University of Texas MD Anderson Cancer Centre, Houston, Texas, U.S.A. p3XFlag-MEF2D was a gift from R. Prywes (Addgene plasmid # 32964).

HA-ERK5 D182A, I115V, D143G, L189F, G199C, S754A, S754D, ΔTAD, Y741*, L664*, A579*, K506* and P435* were made by site-directed mutagenesis using either QuikChange Site-Directed Mutagenesis kit (Agilent) or Q5 Site-Directed Mutagenesis Kit (New England Biolabs) and verified by ABI automated sequencing.

### Luciferase assays

In 96-well tissue culture plates with opaque sides (Thistle Scientific), HEK293 were transfected with pEGFP-MEK5D, pCANHA-ERK5, GAL4-MEF2D, 5XGAL4-Luciferase and CMV-renilla using Lipofectamine 2000 according to the manufacturer’s instructions. Compounds **25** and **26**, AX15836 and BIX02189 were added at concentrations indicated, 4 h post transfection. After a further 20 h, cells were harvested and processed for firefly and renilla luciferase activity using the Promega Dual Luciferase Reporter assay according to the manufacturer’s instructions.

For KLF2-promoter experiments, HEK293 were transfected with pEGFP-MEK5D, pCANHA-ERK5, p3XFlag-MEF2D, luciferase reporter gene driven by the KLF2 promoter (−924 to + 14) and CMV-Renilla using Lipofectamine 2000 according to the manufacturer’s instructions and treated as above.

### Antibody conditions

Antibodies and conditions used are given in Table 1.

**Table 1:** Antibodies and conditions used.

Antibody	Supplier	Catalogue number	Dilution/ diluent	Technique
Anti-MEK5	EMD Millipore	AB3184	1:500/5% milk TBS-T	Immuno-blotting
Anti-phospho-TEY ERK5	Cell Signaling	3371	1:500 / 5% BSA TBS-T	Immuno-blotting
Anti-FLAG M2	Roche/Sigma	F3165	1:2000 / 5% milk TBS-T	Immuno-blotting
Anti-HA (12CA5)	Sigma	11583816001	5 µg/ml in Antibody Dilution Buffer: (1x PBS/1% BSA/0.3% Triton™ X-100)	Immuno-fluorescence
Anti-HA	Babraham Institute Monoclonal Facility	N/A	1:10/5% milk TBS-T	Immuno-blotting
Anti-phospho-T733	Gift from Dr. Atanasio Pandiella, University of Salamanca, Spain.	N/A	1:250/5% milk TBS-T	Immuno-blotting
Anti-phospho-S754	Gift from Dr. Atanasio Pandiella, University of Salamanca, Spain.	N/A	1:250/5% milk TBS-T	Immuno-blotting
Anti-myc (9E10)	Santa Cruz	SC-40	1:1000/5% milk TBS-T	Immuno-blotting
Anti-phospho-T359 RSK	Cell Signaling	8753	1:1000/5% milk TBS-T	Immuno-blotting
Anti-RSK	Cell Signaling	9355	1:1000/5% milk TBS-T	Immuno-blotting
Anti-MEK1/2	Cell Signaling	9122	1:1000/5% milk TBS-T	Immuno-blotting
Anti-Lamin A/C	Santa Cruz	SC-7292	1:500/5% milk TBS-T	Immuno-blotting
Anti-ERK5 (C-7)	Santa Cruz	SC-398015	1:1000/5% milk TBS-T	Immuno-blotting

### Immunoblot analysis

For western blotting, 6 cm dishes (Thermo Scientific) were routinely transfected as described then treated with compound **26**, AX15836, SCH772984 or DMSO at the indicated concentrations and for the indicated times prior to harvesting and lysed to prepare whole-cell extracts in 150 µl TG lysis buffer (20 mM Tris-HCl pH 7.5, 137 mM NaCl, 1 mM, EGTA, 1% v/v Triton X-100, 10% v/v glycerol, 1.5 mM MgCl_2_, 1 mM Na_3_VO_4_, 1 mM PMSF, 10 µg/ml leupeptin, 10 μg/ml aprotinin, 50 mM NaF). Samples were subjected to sodium dodecyl sulfate–polyacrylamide gel electrophoresis (SDS-PAGE) on 10% gels, then transferred to PVDF membranes. Membranes were blocked in 5% milk-TBST and incubated with antibody overnight, shaking at 4 °C. The following day, membranes were washed 3 times with TBS-T, incubated for 30 min–1 h with HRP-secondary antibody and the bands imaged with ECL and film. Films were scanned using an EPSON scanner, and images rotated in Adobe Photoshop Elements and the image cut and pasted into Adobe Illustrator to prepare the figures. Alternatively, blots were incubated with IRDye 680 or 800 anti-mouse or anti-rabbit secondary antibodies and visualised using an Odyssey CLx Li-COR scanner. All uncropped blots and scans are in the Source Data file.

### GST pull-downs

For GST pull-downs, 10 cm dishes (Thermo Scientific) were routinely transfected as described then used to prepare whole-cell extracts in 500 µl TG lysis buffer. Two-hundred microlitres lysate was incubated with 20 µl glutathione sepharose 4B (GE Healthcare) (50% slurry in PBS) at 4 °C end-over-end for 2 h. Glutathione-sepharose conjugates were washed two times with TG lysis buffer, spinning at 3000 rcf for 1 min, then subjected to SDS-PAGE and immune blotting using the antibodies indicated.

### Nuclear-cytosolic fractionation

HeLa cells were seeded in 10 cm dishes (Thermo Scientific) and allowed to settle for 24 h. Cells were then treated with 0.1 µM compound **26**, AX15836 or DMSO control for 30 min. Cells were harvested by trypsinisation and centrifugation and snap frozen. The cell pellets were thawed on ice re-suspended in ice cold buffer A (10 mM Hepes pH 7.9, 1.5 mM MgCl_2_, 10 mM KCl, 0.1 mM EDTA, 1 mM Na_3_VO_4_, 1 mM PMSF, 10 µg/ml leupeptin, 10 μg/ml aprotinin, 50 mM NaF). Cell suspensions were incubated on ice to allow cells to swell. In all, 0.1% NP-40 was added and mixed by gently pipetting up and down. The cell suspension was then passed through a 25 G needle to disrupt the cell membrane. The homogenate was centrifuged at 800 rcf to pellet the nuclei. The cytosolic fraction was transferred to a fresh tube and centrifuged three times at 10,000 rcf and supernatant removed to pellet any remaining nuclei. NaCl was added to a final concentration of 137 mM and glycerol to 100%. The nuclear fraction was re-suspended in buffer A and centrifuged at 800 rcf and washed three times with buffer A to remove any residual cytosol. The nuclei pellet was re-suspended in RIPA buffer (50 mM Tris-HCl pH 8, 150 mM NaCl, 1% v/v Triton X-100, 1% w/v sodium deoxycholate, 0.1% w/v SDS, 1 mM Na_3_VO_4_, 1 mM PMSF, 10 µg/ml leupeptin, 10 μg/ml aprotinin, 50 mM NaF) supplemented with 2.25U benzonase/ml and incubated at 4 °C for 1 h end-over-end and occasionally vortexing. Samples were subjected to SDS-PAGE and immuno-blotted with the antibodies indicated.

### Mass spectrometry

MEF2D bands were cut from Coomassie-stained gels, reduced, carbamidomethylated and digested overnight at 30 °C in 25 mM ammonium bicarbonate containing 10 ng/ml of protease (modified trypsin (Promega) or elastase (Promega)). Phosphopeptides were enriched using titanium dioxide beads (Titansphere, GL Sciences) and glycolic acid as competitor. Enriched phosphopeptides, or unfractionated digests, were analysed by nano liquid chromatography–mass spectrometry (LC-MS/MS) on a QExactive Plus mass spectrometer (Thermo Scientific) fitted with a nanoelectrospray ion-source (Proxeon). Peptides were separated on a reversed-phase column (0.075 × 150 mm; ReproSil-Pur 120 C18-AQ 3 mm) using an acetonitrile gradient (5–40% in 30 min at 300 nl/min) containing 0.1% formic acid. Full scan data were acquired over the m/z range 350–1800 at a nominal resolution setting of 70,000, followed by up to 10 higher-energy collisional dissociation (HCD) spectra at 27% relative collision energy. Analyses of the digests with targeted MS/MS of the identified phosphopeptides were done similarly, but with HCD fragmentation at 35% relative collision energy. Mass spectral data were searched against the human entries of the Uniprot 15.14 database using Mascot software (Matrix Science) and all putative phosphopeptide MS/MS spectra were manually validated. Apparent stoichiometries of phosphorylation were calculated from the peak areas of the extracted ion chromatograms of the phospho- and corresponding nonphosphopeptides in LC-MS runs of unfractionated digests as in ref. ^40^.

(Phospho)peptides were identified from mass spectrometry data using Peaks Studio V7 (Bioinformatics Solutions Inc) and manually validated, while quantitative analyses were done with the aid of Skyline Software (MacCoss Lab, Washington University).

### Molecular dynamics simulations

The structure of ERK5^37^ co-crystallised with the specific inhibitor compound **25** (PDB: 4B99) was used as a starting model for molecular dynamics simulation. All solvent molecules were removed, and compound **25** was extracted from the model. Missing loops were built with MODELLER (v9.19)^75,76^ via UCSF Chimera^77^. Mutants were modelled in UCSF Chimera. Compound **25** was modified in Avogadro (v.1.9)^78^ to create compound **26** using the published structure as a ref. 37, changing the R1 group from a methoxy to ethoxy moiety and adding hydrogen atoms. The ligand was then parameterised using Gasteiger point charges in the AMBER ff99sb^79^ force field for simulation using Antechamber^80^, and prepared for GROMACS using ACPYPE^81^. The complexes were then prepared in GROMACS^82^, parameterised in ff99sb and neutralised with sodium and chloride ions to a final concentration of 0.1 M, prior to being subject to minimisation and equilibration. Briefly, a steepest descent gradient of maximum 5000 steps was used to minimise the complexes, which converged on an *F*_max_ < 1000 kJ/mol/nm in <2000 steps. The complexes were then subject to 200 ps equilibration in the NvT ensemble using a 2 fs timestep and Nose-Hoover temperature coupling at 300 K; all atoms were subject to position restraints. Subsequent equilibration in the NpT ensemble was also conducted under position restraints at a 2 fs timestep, with Parinello-Rahman barostat at 1 bar. Finally, position restraints were released and three parallel production MD simulations were conducted for a 10 ns.

The g_mmpbs^83^ package was used to calculate binding energies between ligand and protein. The final 2 ns of converged simulation time was used for each of the simulations, totalling 201 snapshots from 8–10 ns. The binding energies were converted from kJ/mol to the more commonly used kcal/mol and averaged.

To model AX15836 binding to ERK5, the method outlined above for compound **26** was used and run for 100 ns and the root mean squared fluctuation (RMSF) of each residue was calculated for the period 10–100 ns.

### High-content microscopy: equipment and settings

HeLa cells were seeded in Cell CarrierTM-96 plates (Perkin Elmer) then transfected with pCANHA-ERK5 and pEGFP-MEK5D using Extreme-Gene 9 (Sigma) as indicated. Cells were treated with compound **26** or DMSO at the concentrations and for the indicated times prior to fixing with 4% paraformaldehyde for 30 min at 25 °C. The cells were then blocked with Blocking Buffer (1x PBS/5% goat serum/0.3% Triton™ X-100). Slides were then incubated with anti-HA (12CA5) 1:100 in Antibody Dilution Buffer: (1X PBS/1% BSA/0.3% Triton™ X-100) overnight at 4 °C. Slides were washed 3 times with PBS for 5 min per wash then incubated with 1:1000 donkey-anti-mouse 568 Alexa Fluor (Invitrogen) and 1 µg/ml DAPI. Slides were then washed three times for 5 min per wash and left in PBS for imaging.

Cells were imaged using In Cell 6000 at room temperature, with 20x objective lens acquiring GFP, DAPI and dsRed using the InCell software settings. Single section images of xy planes with pixel dimensions 325 x 325 nm and 16 bit image (2048  × 2048 pixels). The fluorochromes used were 568 Alexa Fluor (Laser: 568 nm, Filter: Texas Red, Excitation max: 578 nm, and Emission max: 603 nm), DAPI (Laser: 405 nm, Filter: DAPI, Excitation max: 350 nm, and Emission max: 470 nm), and GFP (Laser:488 nm, Filter: FITC, Excitation max: 488 nm, and Emission max: 510 nm). Results were analysed using In Cell Analyser Workstation 3.7.2 to determine the level of nuclear and cytosolic ERK5. In the Assay Parameters for Nuclei and Cells, Segmentation of nuclei was set to Top-Hat with a minimum area of 50 µm^2^ and Segmentation of cells to Collar with a radius of 2 µm. The Assay Measures were Nuclei Area, Nuc1/(FormFactor), Nuclei Intensity; Reference, Nuc/Cell Intensity, Nuc Intensity, Cell Intensity; Cell tracking, Label, Linked Track ID; and Filters, threshold, Nuc1/(Form Factor), Nuclei. The Cell-by-Cell data was exported to Microsoft Excel as a.csv file. Transfected cells were identified by having a reading > than 2x background intensity. The % of cells with higher nuclear ERK5 staining to cytosolic ERK5 staining was calculated for each experimental well.

Representative.tiff images we merged in ImageJ and the scale bar added. The images were converted to RGB colour, or the red channel displayed as greyscale alone. The brightness/contrast minimum and maximum was fixed to the same values for each image.

### Statistical analysis

Depending on the scientific question, in particular the number of conditions compared, and the experimental design, independent or paired *t*-tests or one-way or repeated measured analysis of variances (ANOVAs) were run to establish statistical significance (*p* < 0.05). ANOVAs were followed by post-hoc tests with Holm–Sidak correction for multiple comparisons. When the experiments were comparing curves, area under the curves were used for the statistical analyses. Where appropriate, to show significance or not, the *p*-values are displayed on the graphs.

### Reporting summary

Further information on research design is available in the Nature Research Reporting Summary linked to this article.

## Supplementary information

Supplementary Information

Reporting Summary

## Data Availability

The data sets analysed during the current study are available in the following repositories: coordinates for the structures of the ERK5-Cpd 25, ERK5-ATP-MEK5 PB1 domain, and PKA-ATP-PKI complexes were retrieved from the Protein Databank in Europe (PDBe) at https://www.ebi.ac.uk/pdbe/ using the accession codes 4B99, 4IC7 and 4WB5, respectively; protein sequences for human AURKA, AURKB, DCLK1, DLCK2, DCLK3, ERK1, ERK2, ERK3, ERK4, ERK5, ERK6, ERK7, ERK8, LRRK2, PLK1, PLK2, PLK3, PLK4, RIPK1, RIPK2 and RIPK5, and for human, mouse and rat MEF2D were retrieved from the UniProt knowledge Base at https://www.uniprot.org/ using the accession codes O14965, Q96GD4, O15075, Q8N568, Q9C098, P27361, P28482, Q16659, P31152, Q13164, P53778, Q8TD08, Q5S007, P53350, Q9NYY3, Q9H4B4, O00444, Q13546, O43353, Q6XUX3, Q14814, Q63943 and O89038, respectively. The source data underlying Figs. 2a–h, 3d–k, 4a–k, 5a, b, d–i and 6a–c, and Supplementary Figs. S1B–D, S2A–F, S3C, S4C, D, S5, S6, S8A–C are provided as a Source Data file. All the other data supporting the findings of this study are available within the article and its supplementary information files and from the corresponding author on reasonable request.

## Source data

Source Data

## Code availability

All code used to generate and analyse the Molecular Dynamic Simulations can be accessed here https://osf.io/thfx3/
