# Supplementary material for: Paradoxical activation of the protein kinase-transcription factor ERK5 by ERK5 kinase inhibitors
Source: Nat Commun. 2020 Mar 13;11:1383. doi: 10.1038/s41467-020-15031-3 (PMC7069993; doi:10.1038/s41467-020-15031-3)
Supplement: Supplementary file 1 — Supplementary Information [file 41467_2020_15031_MOESM1_ESM.pdf]

Paradoxical activation of the protein kinase-transcription factor ERK5

by ERK5 kinase inhibitors

Lochhead et al

Supplementary Figure 1

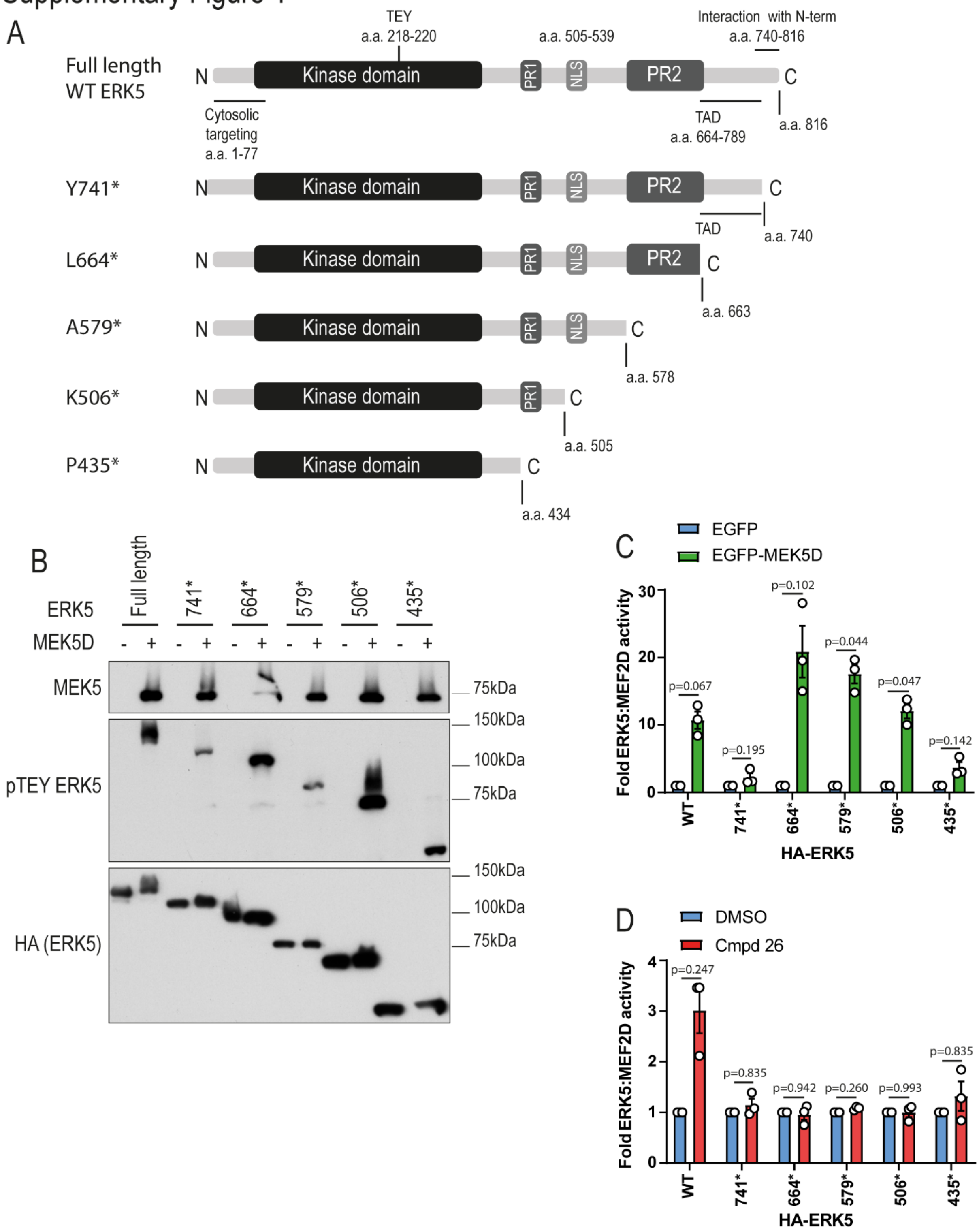

Supplementary Figure 1: The reported minimal TAD mediates the ERK5:MEF2D-transcriptional activity induced by compound 26.

To define which domain in the C-terminus of ERK5 was responsible for cmpd **26**-induced transcriptional activity of ERK5:MEF2D. We generated five successive C-terminal deletion constructs to remove: (i) the region that interacts with the N-terminus (Y741*); (ii) the minimal TAD (L664*); (iii) the proline rich region 2 (A579*) and (iv) the NLS (K506*) and finally (v) the proline rich region 1 (P435*) (supplementary figure 1A). These constructs all expressed in HEK293 cells and were phosphorylated on their TEY motif when MEK5D was co-expressed (supplementary figure 1B). In the ERK5:MEF2D luciferase assay MEK5D increased the activity of L664*, A579*, K506* and P435* but not Y741* (supplementary figure 1C). The Y741* mutant could not be activated by MEK5D, correlating with the level of TEY phosphorylation being reduced compared to wild type. This may suggest that Y741* may not interact as well with MEK5D, or that ERK5 Y741* may be held in an inactive conformation preventing it from interacting with MEF2D (supplementary figure 1B). Cmpd **26** was not able to promote ERK5:MEF2D activity for any of the truncated constructs (supplementary figure 1D). Given that Y741* could not be activated by either MEK5D or cmpd **26**, but that L664* could be activated by MEK5D but not cmpd **26** these results show that the previously reported minimal TAD is required for cmpd **26**-driven ERK5:MEF2D transcriptional activity.

A: Schematic diagram of ERK5 full length and the C-terminal deletion constructs lacking described functional domains: Y741*, termination before the N-terminal interaction domain; L664*, termination before the minimal TAD; A579*, termination before the proline rich region2; K506*, termination before the NLS; P435*, termination before the proline rich region1.

B: HEK293 cells were transfected with HA-ERK5, Flag-MEF2D and either EGFP-MEK5D or EGFP. 24 h post transfection cells were lysed, subjected to SDS-PAGE and immuno-blotted with the antibodies shown. The experiment was repeated 3 times and a representative image is shown. Source data are provided as a Source Data file.

C: HEK293 cells were transfected with GAL4-MEF2D, GAL4:LUC and CMV:Renilla together with either wild type HA-ERK5 (full length), HA-Y741*ERK5, HA-L664*ERK5, HA-A579*ERK5, HA-K506*ERK5 or HA-P435*ERK5 and either EGFP or EGFP-MEK5D. 24 h post-transfection cells were lysed and firefly luciferase activity was measured and normalized to Renilla. The results are presented as the mean of 3 independent experiments ± SEM. Source data are provided as a Source Data file.

D: HEK293 cells were transfected with GAL4-MEF2D, GAL4:LUC and CMV:Renilla together with either wild type HA-ERK5 (full length), HA-Y741* ERK5, HA-L664* ERK5, HA-A579* ERK5, HA-K506* ERK5 or HA-P435* ERK5 and EGFP. 4 h post-transfection cells were treated with either 100nM cmpd **26** or DMSO (control) as indicated. 24 h post-transfection cells were lysed and processed as in **C**.

Supplementary Figure 2

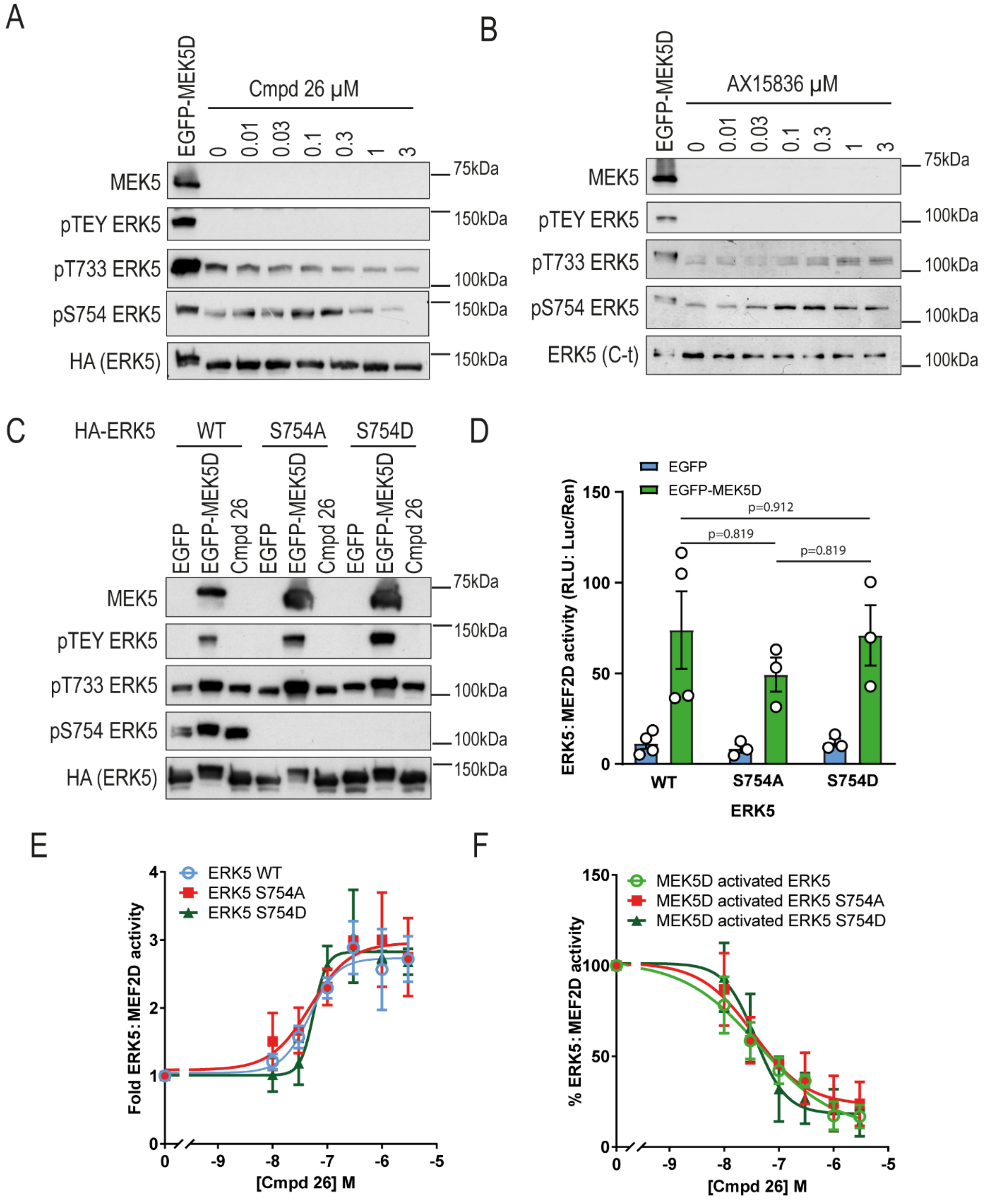

Supplementary Figure 2: ERK5i do not require phosphorylation of the ERK5 C-terminal region to stimulate ERK5:MEF2D driven gene transcription.

Phosphorylation of the ERK5 C-terminus promotes transcriptional activity and occurs either by auto-phosphorylation [11] or by upstream kinases [15] [16, 17]. We used two phospho-specific antibodies to characterise phosphorylation sites on the C-terminus of ERK5; T733 and S754 [16]. As a control, we co-

expressed MEK5D with ERK5; as expected the activation-loop TEY motif was phosphorylated as well as T733 and S754 compared to no MEK5D expression (supplementary figure 2A and B). A basal level of phospho-T733 was detected and decreased as cmpd **26** concentration increased, for AX15836, T733 phosphorylation increased slightly with increasing concentrations. In contrast, ERK5 S754 phosphorylation increased with increasing concentrations of cmpd **26** and AX15836, peaking at 300nM. To test the involvement of S754 phosphorylation we produced mutants of ERK5 where S754 was mutated to alanine, to prevent phosphorylation, or aspartic acid, to mimic phosphorylation. Immuno-blotting confirmed that neither ERK5 S754A or S754D was detected with the phospho-S754 antibody following MEK5D expression or cmpd **26** treatment (supplementary figure 2C). These mutants were then tested in the ERK5:MEF2D luciferase assay in the presence of cmpd **26**. Neither of these mutations affected the ability of ERK5 to induce MEF2D transcriptional activity in the luciferase assay compared to wild type (supplementary figure 2D). Wild type, S754A and S754D ERK5 all exhibited the same paradoxical activation of ERK5:MEF2D luciferase assay by cmpd **26** (supplementary figure 2E), and residual ERK5:MEF2D activity was detected with wild type, S754A and S754D mutants when ERK5 was activated by MEK5D and incubated with cmpd **26** (supplementary figure 2F). Therefore, although S754 becomes phosphorylated following cmpd **26** treatment, this phosphorylation was not required for cmpd **26** induction of ERK5:MEF2D transcriptional activity.

A and **B:** HEK293 cells were transfected with HA-ERK5 and either EGFP-MEK5D or EGFP. 4 h post-transfection cells were treated with either DMSO (control), cmpd 26 (**A**) or AX15836 (**B**) at the concentrations indicated. 24 h post transfection cells were lysed, subjected to SDS-PAGE and immuno-blotted with the antibodies shown. The experiment was repeated 3 times and a representative image is shown. Source data are provided as a Source Data file.

C: HEK293 cells were transfected with either HA-ERK5, HA-S754A ERK5, or HA-S754D ERK5 and either EGFP-MEK5D or EGFP. 4 h post-transfection cells were treated with either DMSO (control) or 100nM cmpd 26. 24 h post transfection cells were lysed, subjected to SDS-PAGE and immuno-blotted with the antibodies shown. The experiment was repeated 3 times and a representative image is shown. Source data are provided as a Source Data file.

D: HEK293 cells were transfected with GAL4-MEF2D, GAL4:LUC and CMV:Renilla together with either wild type HA-ERK5, HA-S754A ERK5 or HA-S754D ERK5 and either EGFP or EGFP-MEK5D. 24 h post-transfection cells were lysed and firefly luciferase activity was measured and normalized to Renilla. The results are presented as the mean of at least 3 independent experiments ± SEM. Source data are provided as a Source Data file.

E: HEK293 cells were transfected with GAL4-MEF2D, GAL4:LUC and CMV:Renilla together with either wild type HA-ERK5, HA-S754A ERK5 or HA-S754D ERK5 and EGFP. 4 h post-transfection cells were treated with either DMSO (control) or cmpd 26 at the concentrations indicated. 24 h post-transfection cells were lysed and firefly luciferase activity was measured and normalized to Renilla.

The results are presented as the mean of 3 independent experiments ± SEM. Source data are provided as a Source Data file.

F: HEK293 cells were transfected with GAL4-MEF2D, GAL4:LUC and CMV:Renilla together with either wild type HA-ERK5, HA-S754A ERK5, HA-S754D ERK5 and EGFP-MEK5D. Cells were then treated with either DMSO (control) or cmpd 26 at the concentrations indicated. 24 h post-transfection cells were lysed and processed as in **E**.

Supplementary Figure 3

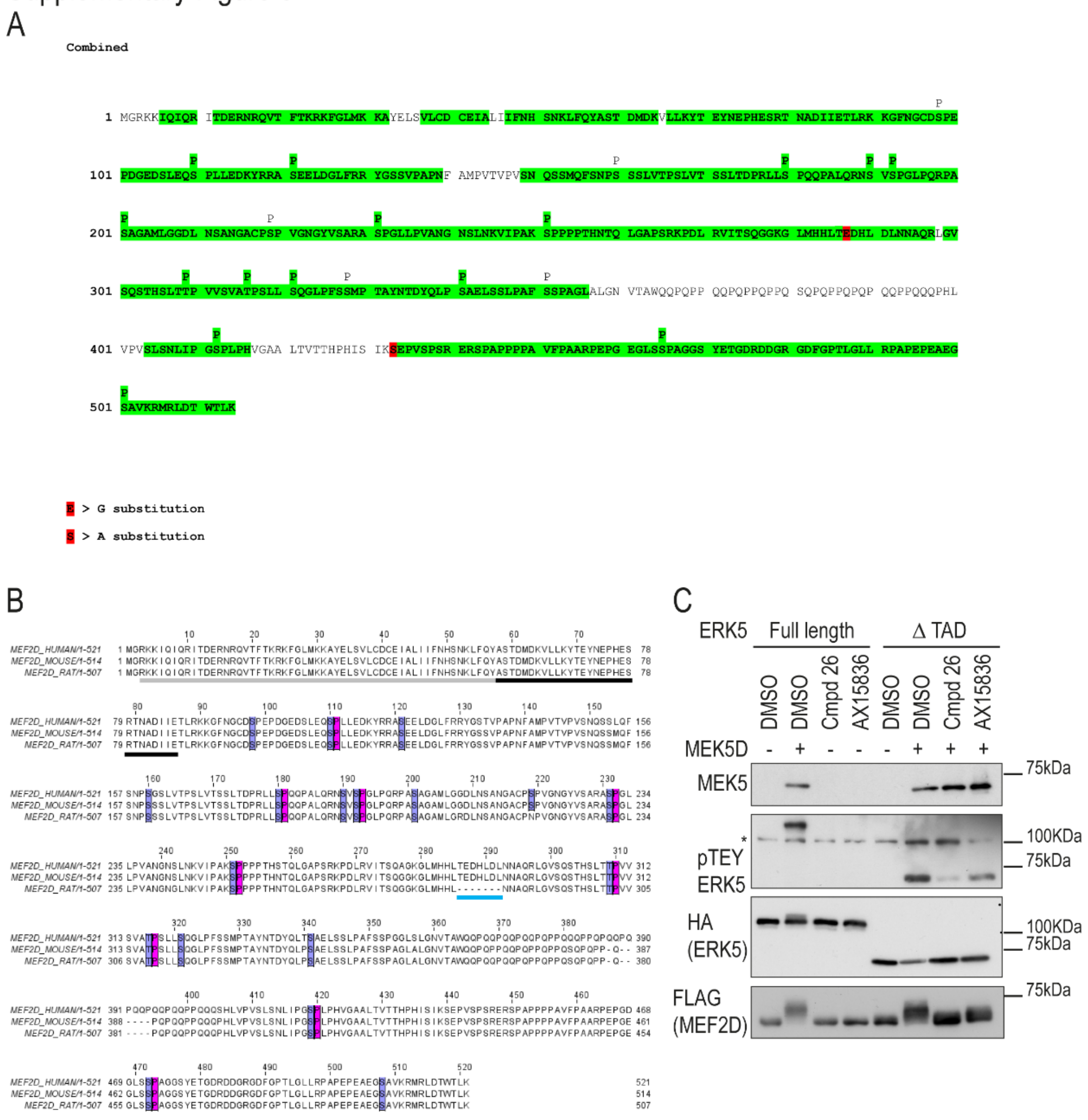

Supplementary Figure 3: ERK5i do not require phosphorylation of MEF2D to stimulate ERK5:MEF2D driven gene transcription.

ERK5 has been shown to promote MEF2D transcriptional activity by phosphorylating S180 [14]. Additional MEF2D phosphorylation sites have been reported on phosphosite.org. To address which of

these were driven by ERK5 we co-expressed ERK5 and (murine) MEF2D in the presence or absence of MEK5D. We identified 18 phosphorylation sites: S98, S110, S121, S160, S180, S190, S192, S201, S219, S231, S251, T309, T316, S321, S341, S412 and S465 (*Mus musculus* numbering) all of which are conserved in human and rat (supplementary figure 3A and B). From assessing the apparent stoichiometry, S192 was constitutive, but S121, S180, S192, S231, S341 and S465 all increased when active ERK5 was co-expressed (supplementary table 1). For the other sites identified, insufficient data was obtained to determine their apparent stoichiometry, but peptides containing S190, S192, S201 and S341 were only detected in samples prepared from co-expression of active ERK5 with MEF2D. Interestingly nine of these sites were proline directed (Ser-Pro), the preferred consensus sequence for MAP kinases. Consistent with phosphorylation detected by mass spectrometry we observed a band-shift in MEF2D migration on SDS-PAGE when ERK5 was activated by MEK5D compared to non-active ERK5. Cmpd **26** and AX15839 treatment did not promote a MEF2D band-shift suggesting that phosphorylation of MEF2D was not promoted in these conditions. Cmpd **26** and AX15836 were active in this experiment as they blocked the MEF2D band-shift that was observed when ERK5ΔTAD was activated by MEK5D (supplementary figure 3C).

A: Total sequence coverage of MEF2D from the trypsin and elastase peptide mapping experiments and phosphorylation sites identified by MS/MS. HEK293 cells were transfected with HA-ERK5, Flag-MEF2D and either EGFP-MEK5D or EGFP. 24 h post transfection cells were lysed and Flag-MEF2D isolated by immuno-precipitation, subjected to SDS-PAGE and visualised with SimplyBlue safe stain. Flag-MEF2D was excised, protein digested and the phospho-peptides and phosphorylation sites were identified by MS/MS. Probable single amino-acid substitutions detected (compared to the UniProtID mouse MEF2D Q63943) are highlighted in red. “P” above an S/T residue indicates a site for which mass spectral evidence of phosphorylation was obtained, with the high confidence phosphorylation sites highlighted in green.

B: Alignment of human, mouse and rat MEF2D showing phosphorylation sites identified by MS/MS. Amino acids highlighted in blue were phosphorylated; prolines highlighted in magenta are +1 to phosphorylated serine or threonine. The putative MADS-box and Mef2-type DNA-binding domains, and the beta (transcriptional enhancer) domain of human MEF2D are indicated below the sequence by grey, black and cyan bars respectively. Sequences were retrieved from UniProt with the following UniProtID: human MEF2D Q14814, mouse MEF2D Q63943, and rat MEF2D O89039.

C: HEK293 cells were transfected as in **A**. 4 h post transfection cells were treated with either cmpd **26** (100 nM), AX15836 (100 nM) or DMSO. 24 h post transfection cells were lysed, subjected to SDS-PAGE and immuno-blotted with the antibodies shown. * non-specific band. The experiment was repeated 3 times and a representative image is shown. Source data are provided as a Source Data file.

Supplementary Table 1: ERK5 Mediated Phosphorylation Sites on MEF2D

Phosphory-lation Site (Mouse)	Peptide	Phospho-peptide detected when ERK5 expressed	Area Non-Phosphorylated		Area Phosphorylated		Area Ratio ERK5/ Control	Norma-lised Area	Normalised Ratio ERK5/ control	Apparent Stoichio-metry (%)
			Control	Plus ERK5	Control	Plus ERK5				
**98**	**LRKKGFNGCDS[+80]PEPDGEDSL**	ND	ND	ND	ND	ND				NK
**110**	**EQS[+80]PLLEDKYRRA**	YB	ND		1711328					NK
				ND		13709558	8.0			NK
**121**	**RAS[+80]EELDGLFR**	YB	7069209		1549569			0.2		18
				15738058		15356584	9.9	1.0	4.5	49
**160**	**SMQFSNPS[+80]SSLVTPS**	YB	ND		402712					NK
				ND		336640	0.8			NK
**180**	**SLTDPRLLS[+80]PQQPA**	YB	24031278		2894330			0.1		11
				19512440		43449776	15.0	2.2	18.5	69
**192**	**RNSVS[+80]PGLPQRPA**	YB	1185875		2804591			2.4		70
				474481		6725050	2.4	14.2	6.0	93
**190, 192**	**RNS[+80]VS[+80]PGLPQRPA**	YB	1185875		9267			0.0		0
				474481		20210	2.2	0.0	5.5	0
**192, 201**	**RNSVS[+80]PGLPQRPAS[+80]A**	YB	ND		9737					NK
				ND		6463140	663.8			NK
**190, 192, 201**	**RNS[+80]VS[+80]PGLPQRPAS[+80]A**	YO	ND	ND	ND	195208				NK
**192**	**SVS[+80]PGLPQRPA**	YB	75697888		6089303			0.1		7
				22244212		18653562	3.1	0.8	10.4	46
**201**	**SVSPGLPQRPAS[+80]A**	YB	ND		381380					NK
				ND		7729614	20.3			NK
**192, 201**	**SVS[+80]PGLPQRPAS[+80]A**	YB	ND		96632					NK
				ND		12446690	128.8			NK
**219**	**GAMLGGDLNSANGACPS[+80]PV**	ND	ND	ND	ND	ND				NK
**231**	**RAS[+80]PGLLPV**	YB	53838904		9081940			0.2		14
				62351184		127391520	14.0	2.0	12.1	67
**251**	**ANGNSLNKVIPAKS[+80]PPPPTHNT**	YB	ND		505901					NK
				ND		5298572	10.5			NK
**309**	**THSLTT[+80]PVVS**	ND	ND	ND	ND	ND				NK
**309, 316**	**THSLTT[+80]PVVSVAT[+80]PS**	ND	ND	ND	ND	ND				NK
**321**	**LLS[+80]QGLPFSSMPT**	ND	ND	ND	ND	ND				NK
**341**	**AYNTDYQLPS[+80]AELS**	YO	162070		ND		0			0
				1105885		2215475	2			67
**341**	**DYQLPS[+80]AELS**	YB	4186977		17938			0		0
				611075		5672230	316.2	9.3	2166.6	90
**412**	**NLIPGS[+80]PLPH**	ND	ND	ND	ND	ND				NK
**465**	**ARPEPGEGLSS[+80]PAGG**	YB	11549656		14232626			1.2		55
				5804519		105884720	7.4	18.2	14.8	95

**501**	**LLRPAPEPE AEGS[+80] AV**	ND	ND	ND	ND	ND	NK

NK: not known, ND: not determined, YB: Yes in samples with and without ERK5 (both), YO: yes in sample with ERK5 only.

Mass spectral peak areas of MEF2D phospho- and corresponding non-phospho-peptides (where detected) were obtained from targeted LC-MS/MS analyses. They were calculated for each peptide as the sum of the peak areas of diagnostic MS2 fragment ions, using Skyline software (MacCoss Lab, Univ Washington). “Normalised area” is the area of a phosphopeptide divided by the area of the corresponding non-phosphopeptide. “Normalised ratio” is the ratio of the normalised areas for a phosphopeptide in ERK5 *vs* control sample.

Supplementary Figure 4

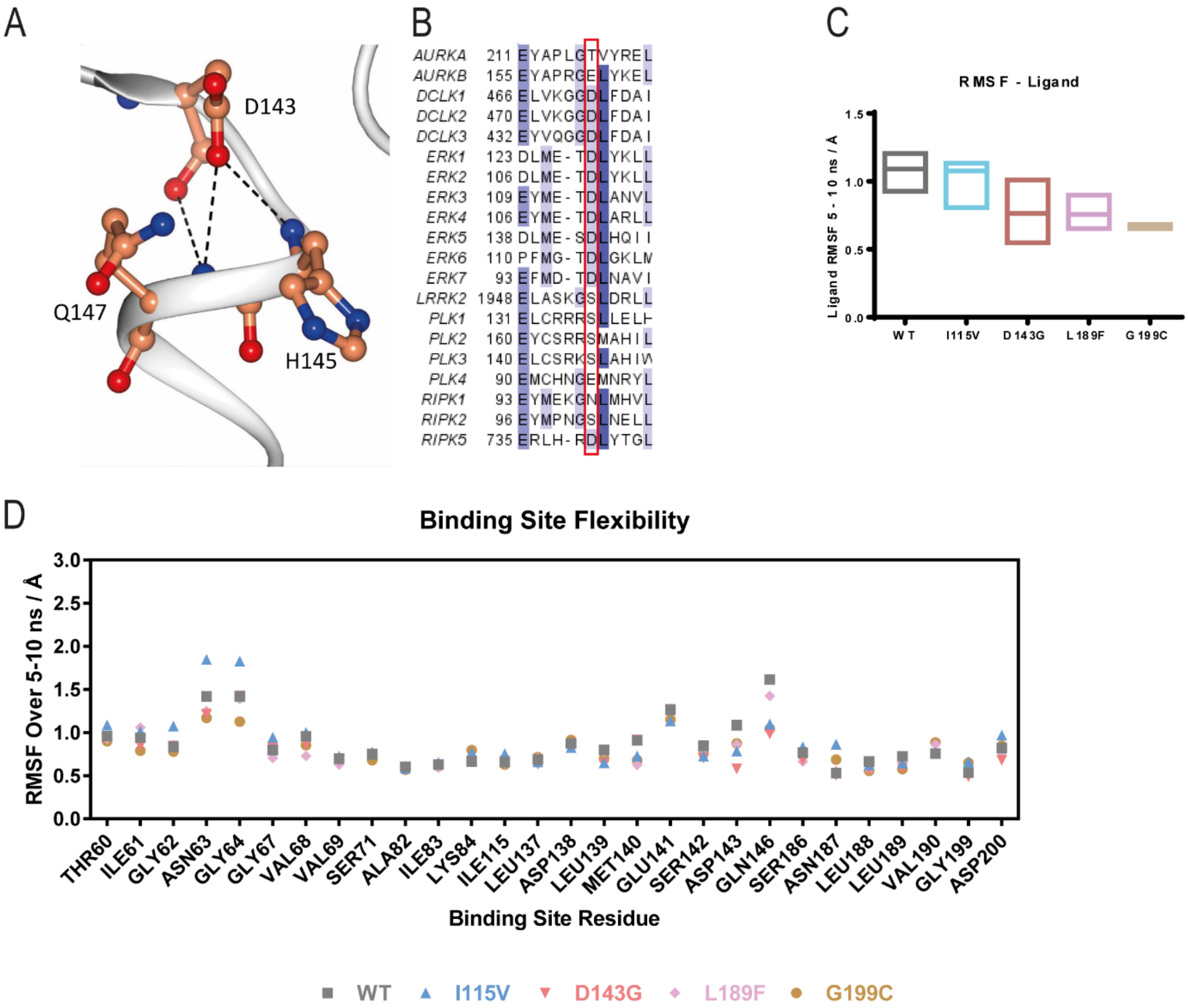

Supplementary Figure 4: Additional information for the generation of compound 26 resistant kinase active mutants of ERK5.

A: The side-chain of ERK5 D143 forms a hydrogen bonding network or 'helix cap' with the backbone NH groups of H145 and Q147, which stabilises the short αD helix. Figure prepared using CCP4MG [83].

B: Structure-guided sequence alignment of the region surrounding ERK5 D143 across a subset of kinases shows aspartate to be completely conserved at this position within the ERK family, and more generally conserved as an amino acid capable of forming a helix cap. The loop immediately preceding D143 is one residue shorter in members of the ERK family than in many other kinases. Comparison of sequences for kinases shown to bind cmpd **26** (AURKA, DCLK3, ERK5, LRRK2, PLK1, PLK4 and RIPK5), with a selection of those that do not (AURKB, DCLK1, DCLK2, ERK1, ERK2, ERK3, ERK4, ERK6, ERK7, PLK2, PLK3, RIPK1 and RIPK2) shows that the identity of the amino acid at this position does not appear to be correlated with cmpd **26** binding. Sequences were retrieved from the

UniProt database [84], aligned using T-Coffee [87], and the alignment coloured by sequence identity (where darker blue indicates higher conservation) and annotated using Jalview [86].

C: Root mean square fluctuation (RMSF) of cmpd **26** over the final 5 ns of three independent molecular dynamics simulations shown as box plot with mean, demonstrating little movement in the bound ligand in silico for each of the WT and mutant ERK5 models. Source data are provided as a Source Data file.

D: Average root mean square fluctuation (RMSF) of residues in the active site of ERK5 over the final 5 ns of three independent molecular dynamics simulations, demonstrating very little variance between mutants bound to cmpd **26**. Residues N63 and G64 show increased flexibility in I115V suggestive of freer P-loop movement however this is not statistically significant. Source data are provided as a Source Data file.

Supplementary Figure 5

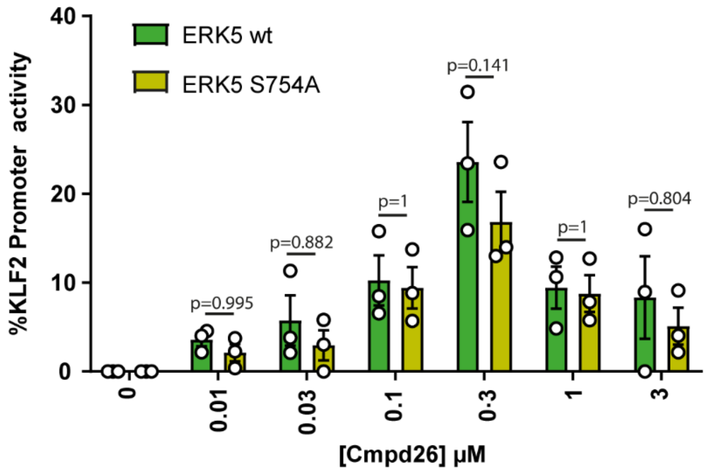

Supplementary Figure 5: Compound 26 induction of the KLF2 promoter is independent of ERK5 S754 phosphorylation.

HEK293 cells were transfected with KLF2:LUC and CMV:Renilla together with FLAG-MEF2D and either wild type HA-ERK5 or HA-ERK5 S754A, and either EGFP-MEK5D or EGFP (control) as indicated, 4h post transfection the cells were treated with Cmpd 26 at the concentrations indicated. 24 h post-transfection cells were lysed and firefly luciferase activity was measured and normalized to Renilla. The results are presented as the mean of 3 independent experiments ± SEM. Source data are provided as a Source Data file.

Supplementary Figure S6

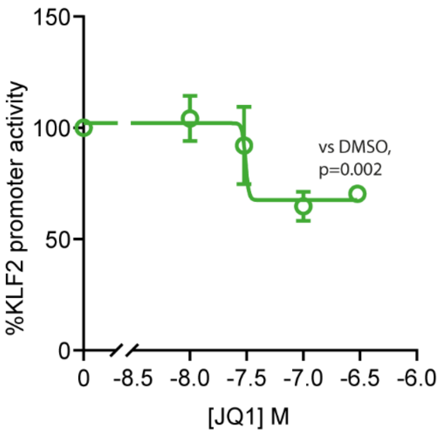

Supplementary Figure 6: The KLF2 promoter is sensitive to JQ1.

HEK293 cells were transfected with KLF2:LUC and CMV:Renilla together with FLAG-MEF2D and wild type HA-ERK5 and either EGFP-MEK5D or EGFP (control) as indicated, 4h post transfection the cells were treated with JQ1 at the concentrations indicated. 24 h post-transfection cells were lysed and firefly luciferase activity was measured and normalized to Renilla. The results are presented as the mean of 3 independent experiments ± SEM. Source data are provided as a Source Data file.

Supplementary Figure 7

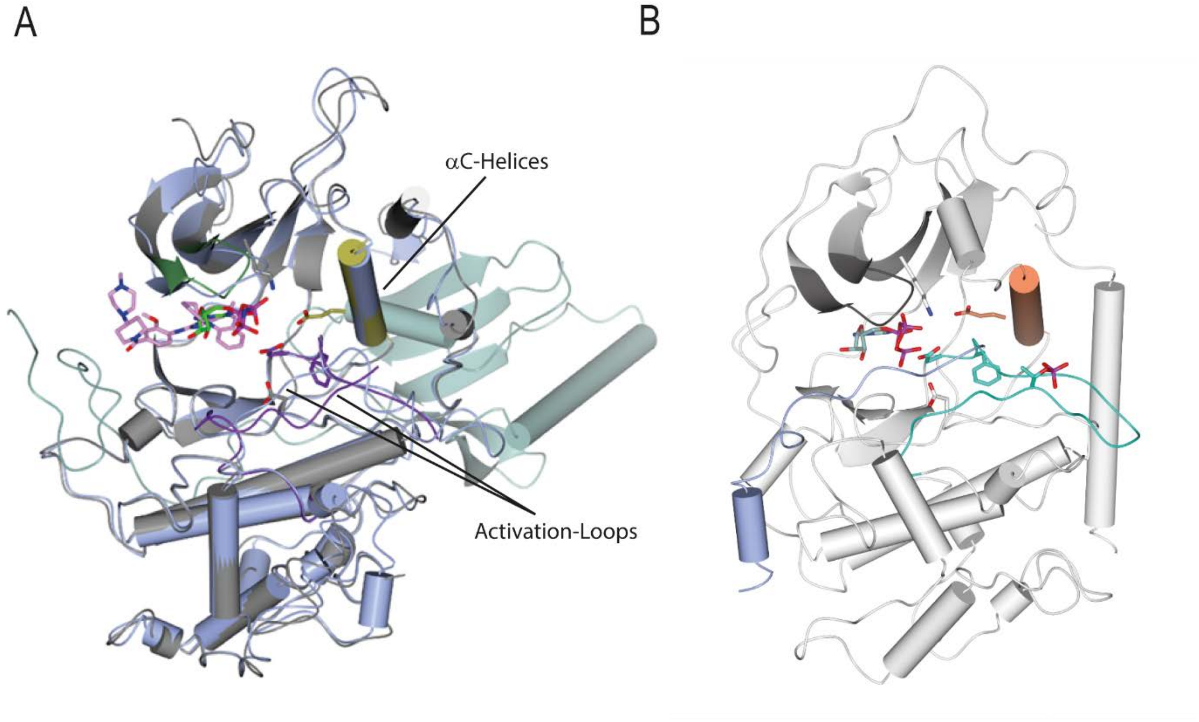

Supplementary Figure 7: Comparison of the structures of ERK5 in complex with ATP and the MEK5 PB1 domain, ERK5 in complex with compound 25 and the structure of PKA in complex with ATP and PKI

A: An overlay of the structures of non-phosphorylated ERK5 in complex with ATP and the MEK5 PB1 domain (PDB 4IC7; ERK5 in ice-blue, MEK5 in pale green and carbon atoms of ATP in green) and ERK5 in complex with compound **25** (PDB 4B99; protein coloured grey and carbon atoms of compound **25** in pink) shows that in the presence of compound **25,** ERK5 adopts a conformation similar to that observed when bound to substrates, with the exception of the activation loop (highlighted in purple) which is partially ordered.

B: Comparison of the ERK5: compound **25** structure with that of Protein Kinase A (PKA) in complex with ATP (carbon atoms in green) and PKI (ice-blue) (PDB 4WB5) also suggests that the former possesses many features consistent with an active kinase structure. Namely, (i) the N- and C-lobes of the kinase domain are partially closed over the ATP-binding site, (ii) the αC-helix (gold in ERK5 and coral in PKA) is rotated in toward the ATP-binding site, (iii) the catalytic aspartate is oriented for phospho-transfer (side-chain shown with carbon atoms in grey), and (iv) the conserved DFG motif at the N-terminus of the activation loop (side-chains shown in purple in ERK5, cyan in PKA) is in the “in” conformation. Figures prepared using CCP4MG [83].

Supplementary Figure 8

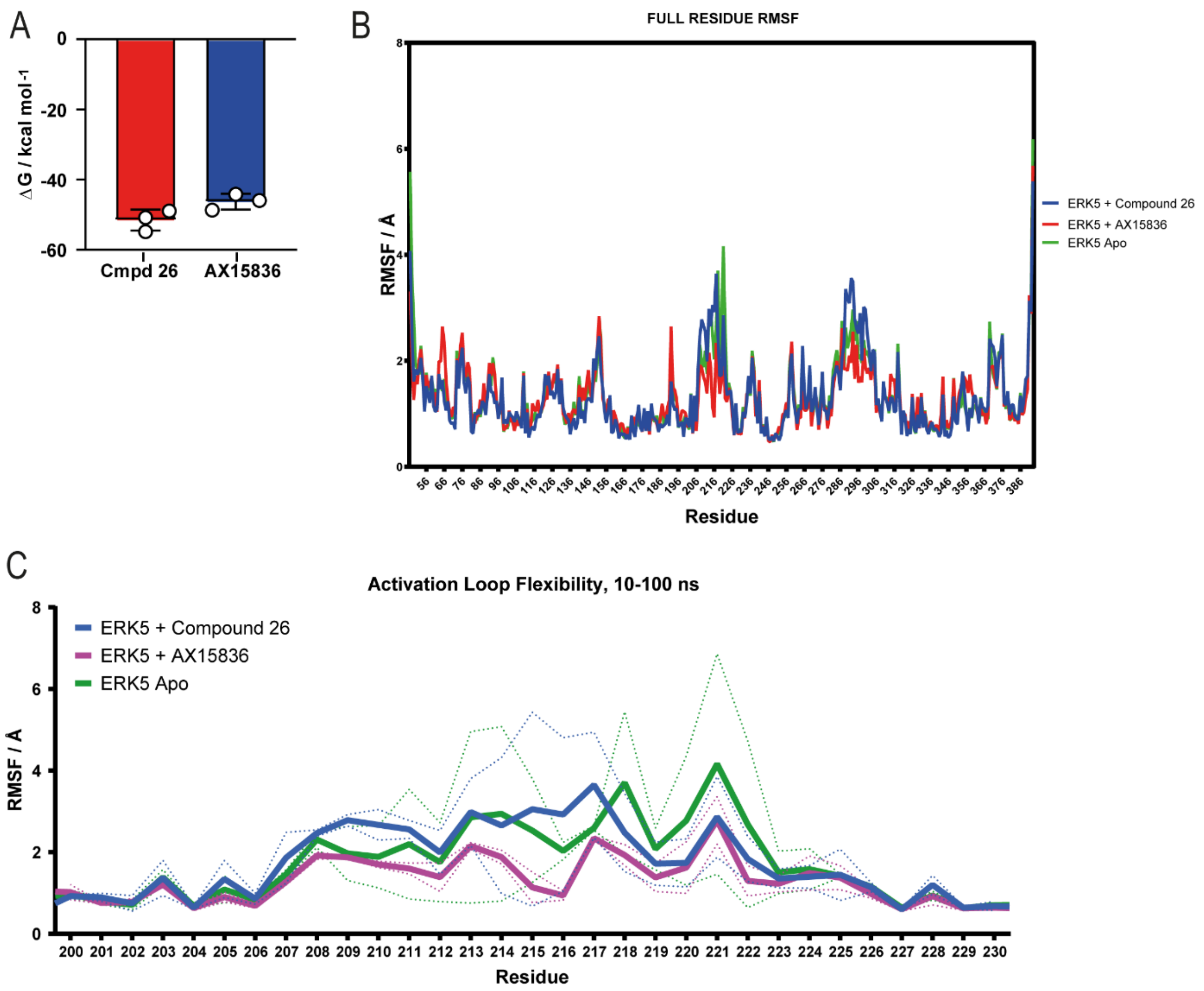

Supplementary Figure 8: In silico comparison of AX15836 bound versus unbound ERK5 shows a reduction in the flexibility of the ERK5 activation-loop.

A: In silico calculation of the binding energy of cmpd 26 and AX15836 to ERK5. Source data are provided as a Source Data file.

B and **C:** In silico analysis of cmpd 26 bound, AX15836 bound and unbound ERK5 shows that AX15836 causes a significant reduction in the flexibility of amino acid 292 (**B**) and 215 and 216 in the activation loop (**B** and **C**). Source data are provided as a Source Data file.
