# Supplementary material for: Paradoxical activation of the protein kinase-transcription factor ERK5 by ERK5 kinase inhibitors
Source: Nat Commun. 2020 Mar 13;11:1383. doi: 10.1038/s41467-020-15031-3 (PMC7069993; doi:10.1038/s41467-020-15031-3)
Supplement: Supplementary file 2 — Reporting Summary [file 41467_2020_15031_MOESM2_ESM.pdf]

nature research

Corresponding author(s): Pamela Lochhead and Simon Cook
Last updated by author(s): Jan 22, 2020

Reporting Summary

Nature Research wishes to improve the reproducibility of the work that we publish. This form provides structure for consistency and transparency in reporting. For further information on Nature Research policies, see Authors & Referees and the Editorial Policy Checklist.

Statistics

For all statistical analyses, confirm that the following items are present in the figure legend, table legend, main text, or Methods section.

n/a	Confirmed	
☐	☒	The exact sample size (*n*) for each experimental group/condition, given as a discrete number and unit of measurement
☐	☒	A statement on whether measurements were taken from distinct samples or whether the same sample was measured repeatedly
☐	☒	The statistical test(s) used AND whether they are one- or two-sided<br>*Only common tests should be described solely by name; describe more complex techniques in the Methods section.*
☒	☐	A description of all covariates tested
☒	☐	A description of any assumptions or corrections, such as tests of normality and adjustment for multiple comparisons
☐	☒	A full description of the statistical parameters including central tendency (e.g. means) or other basic estimates (e.g. regression coefficient) AND variation (e.g. standard deviation) or associated estimates of uncertainty (e.g. confidence intervals)
☐	☒	For null hypothesis testing, the test statistic (e.g. *F*, *t*, *r*) with confidence intervals, effect sizes, degrees of freedom and *P* value noted<br>*Give P values as exact values whenever suitable.*
☒	☐	For Bayesian analysis, information on the choice of priors and Markov chain Monte Carlo settings
☒	☐	For hierarchical and complex designs, identification of the appropriate level for tests and full reporting of outcomes
☒	☐	Estimates of effect sizes (e.g. Cohen's *d*, Pearson's *r*), indicating how they were calculated

Our web collection on statistics for biologists contains articles on many of the points above.

Software and code

Policy information about availability of computer code

Data collection	No software was used.
Data analysis	Protein structure figures were prepared using CCP4MG v2.10.10<br>Protein sequence alignments were generated using T-COFFEE Version_11.00.8cbe486 (2014-08-12 22:05:29 - Revision 8cbe486 - Build 477) or ClustalOmega v1.2.2<br>Protein sequence aligmnments were annotated using Jalview v2.10.5<br>2D depictions of compounds used in this study were generated using ChemDraw v16.0

For manuscripts utilizing custom algorithms or software that are central to the research but not yet described in published literature, software must be made available to editors/reviewers. We strongly encourage code deposition in a community repository (e.g. GitHub). See the Nature Research guidelines for submitting code & software for further information.

Data

Policy information about availability of data

All manuscripts must include a data availability statement. This statement should provide the following information, where applicable:

- Accession codes, unique identifiers, or web links for publicly available datasets
- A list of figures that have associated raw data
- A description of any restrictions on data availability

The datasets analysed during the current study are available in the following repositories: coordinates for the structures of the ERK5-Cpd 25, ERK5-ATP-MEK5 PB1 domain, and PKA-ATP-PKI complexes were retrieved from the Protein Databank in Europe (PDBe) at https://www.ebi.ac.uk/pdbe/ using the accession codes 4B99 [http://dx.doi.org/10.2210/pdb4B99/pdb], 4IC7 [http://dx.doi.org/10.2210/pdb4IC7/pdb] and 4WB5 [http://dx.doi.org/10.2210/pdb4WB5/pdb], respectively; protein sequences for human AURKA, AURKB, DCLK1, DLCK2, DCLK3, ERK1, ERK2, ERK3, ERK4, ERK5, ERK6, ERK7, ERK8, LRRK2, PLK1, PLK2, PLK3, PLK4, RIPK1, RIPK2 and RIPK5, and for human, mouse and rat MEF2D were retrieved from the UniProt knowledge Base at https://www.uniprot.org/ using the accession codes O14965 [https://www.uniprot.org/uniprot/O14965], Q96GD4 [https://www.uniprot.org/uniprot/Q96GD4], O15075 [https://www.uniprot.org/uniprot/O15075], Q8N568

[https://www.uniprot.org/uniprot/Q8N568], Q9C098 [https://www.uniprot.org/uniprot/Q9C098], P27361 [https://www.uniprot.org/uniprot/P27361], P28482 [https://www.uniprot.org/uniprot/P28482], Q16659 [https://www.uniprot.org/uniprot/Q16659], P31152 [https://www.uniprot.org/uniprot/P31152], Q13164 [https://www.uniprot.org/uniprot/Q13164], P53778 [https://www.uniprot.org/uniprot/P53778], Q8TD08 [https://www.uniprot.org/uniprot/Q8TD08], Q5S007 [https://www.uniprot.org/uniprot/Q5S007], P53350 [https://www.uniprot.org/uniprot/P53350], Q9NYY3 [https://www.uniprot.org/uniprot/Q9NYY3], Q9H4B4 [https://www.uniprot.org/uniprot/Q9H4B4], O00444 [https://www.uniprot.org/uniprot/O00444], Q13546 [https://www.uniprot.org/uniprot/Q13546], O43353 [https://www.uniprot.org/uniprot/O43353], Q6XUX3 [https://www.uniprot.org/uniprot/Q6XUX3], Q14814 [https://www.uniprot.org/uniprot/Q14814], Q63943 [https://www.uniprot.org/uniprot/Q63943] and O89038 [https://www.uniprot.org/uniprot/O89038], respectively.
The source data underlying Figs 2A-H, 3D-K, 4A-K, 5A, B, D-I and 6A-C, and Supplementary Figs S1B-D, S2A-F, S3C, S4C and D, S5, S6, S8A-C are provided as a Source Data file. All the other data supporting the findings of this study are available within the article and its supplementary information files and The datasets generated during the current study are available from the corresponding author on reasonable request. A reporting summary for this article is available as a Supplementary Information file.

Field-specific reporting

Please select the one below that is the best fit for your research. If you are not sure, read the appropriate sections before making your selection.

☒ Life sciences ☐ Behavioural & social sciences ☐ Ecological, evolutionary & environmental sciences

For a reference copy of the document with all sections, see nature.com/documents/nr-reporting-summary-flat.pdf

Life sciences study design

All studies must disclose on these points even when the disclosure is negative.

Sample size	Based on previous experience, each was conducted at least 3 times.
Data exclusions	Data was excluded when deemed to be too high or too low compared to other values. The excluded values are shown in the source data files for figures 3D, 4J and 5F.
Replication	Each experiment was conducted 3 times and reproducible results were obtained.
Randomization	For each experiment positive and negative controls were conducted on the same 96 well plate for luciferase assays and high content microscopy, or in the same set of dishes for immuno-blots.
Blinding	Blinding was not relevant to this study and for high throughput and content experiments data collection was automated.

Reporting for specific materials, systems and methods

We require information from authors about some types of materials, experimental systems and methods used in many studies. Here, indicate whether each material, system or method listed is relevant to your study. If you are not sure if a list item applies to your research, read the appropriate section before selecting a response.

Materials & experimental systems

n/a	Involved in the study	
☐	☒	Antibodies
☐	☒	Eukaryotic cell lines
☒	☐	Palaeontology
☒	☐	Animals and other organisms
☒	☐	Human research participants
☒	☐	Clinical data

Methods

n/a	Involved in the study	
☒	☐	ChIP-seq
☒	☐	Flow cytometry
☒	☐	MRI-based neuroimaging

Antibodies

Antibodies used

anti-MEK5 EMD Millipore AB3184 Lot:2913871
anti-phospho TEY ERK5 Cell Signaling 3371 Lot:10
anti-FLAG Roche/Sigma F3165 M2 Lot:not known
anti-HA Sigma 11583816001 12CA5 Lot:not known (immuno-fluorescence)
anti-HA Babraham Institute Monoclonal Facility (immuno-blotting)
anti-phosphoT733 ERK5 and anti-phospho S754 ERK5 gift from Dr. Atanasio Pandiella, University of Salamanca, Spain.
anti-myc (9E10) Santa Cruz SC-40 Lot#J0115
anti-phospho T359 RSK Cell Signaling 8753 Lot:5
anti-RSK Cell Signaling 9355 Lot:5
anti-MEK1/2 Cell Signaling 9122 Lot:14

	anti-Lamin A/C (636) Santa Cruz SC-7292 Lot#H1903
Validation	anti-MEK5: http://www.merckmillipore.com/GB/en/product/Anti-MEK5-Antibody,MM_NF-AB3184?bd=1#documentation and this manuscript.<br>anti-phospho TEY ERK5: https://www.cellsignal.co.uk/products/primary-antibodies/phospho-erk5-thr218-tyr220-antibody/3371?site-search-type=Products&N=4294956287&Ntt=erk5+3371&fromPage=plp&_requestid=156436 and this manuscript.<br>anti-FLAG: https://www.sigmaaldrich.com/catalog/product/sigma/f1804?lang=en&region=GB&utm_medium=cpc&utm_source=bing&utm_term=f3165&utm_campaign=Sigma%20Position%20Support%20Global%20(Bing%20ebizpfs)&utm_content=SIGMA/F1804 and this manuscript and this manuscript.<br>anti-HA Roche: https://www.sigmaaldrich.com/catalog/product/roche/roaha?lang=en&region=GB, https://www.sigmaaldrich.com/catalog/product/roche/roaha?lang=en&region=GB and this manuscript.<br>anti-HA Babraham Institute Monoclonal Facility: this manuscript, PMID: 21924351, PMID: 19249353 and PMID: 17525735<br>anti-phosphoT733 ERK5 and anti-phospho S754 ERK5: this manuscript, PMID: 20736311 and PMID: 21924351.<br>anti-myc (9E10): https://www.scbt.com/p/c-myc-antibody-9e10<br>anti-phospho T359: https://www.cellsignal.com/products/primary-antibodies/phospho-p90rsk-thr359-d1e9-rabbit-mab/8753<br>anti-RSK: https://www.cellsignal.co.uk/products/primary-antibodies/rsk1-rsk2-rsk3-32d7-rabbit-mab/9355<br>anti-MEK1/2: https://www.cellsignal.co.uk/products/primary-antibodies/mek1-2-antibody/9122<br>anti-Lamin A/C (636): https://www.scbt.com/p/lamin-a-c-antibody-636

Eukaryotic cell lines

Policy information about cell lines

Cell line source(s)	HEK293 cells: Babraham Institute.<br>HeLa: Northern Institute for Cancer Research, Newcastle University.
Authentication	HEK293 cells were purchased from ATCC.<br>HeLa cells were purchased from ATCC.
Mycoplasma contamination	The cells tested negative for mycoplasma.
Commonly misidentified lines<br>(See ICLAC register)	HEK293 cells: these cells were used as they transfect easily and express recombinant protein well and therefore ideally suited for this work.
